# Integration of transcriptomics and metabolomics reveals toxicological mechanisms of ZhuRiHeng drop pill in the 180-day repeated oral toxicity study

**DOI:** 10.3389/fphar.2024.1333167

**Published:** 2024-03-15

**Authors:** Qian Zhang, Fang Wang, Jing Liu, Jun Li, Wei Zhang, Shengsang Na, Jingkun Lu, Yuewu Wang

**Affiliations:** ^1^ Inner Mongolia Key Laboratory of Chinese and Mongolian Medicine, Inner Mongolia Medical University, Hohhot, China; ^2^ College of Pharmacy, Inner Mongolia Medical University, Hohhot, China; ^3^ School of Basic Medicine, Inner Mongolia Medical University, Hohhot, China

**Keywords:** traditional Mongolian medicinal preparation, acute toxicity study, 180-day repeated oral toxicity study, metabolomic and transcriptomic profiling, toxicological mechanisms

## Abstract

**Background:** ZhuRiHeng Drop Pill (ZRH) is a traditional Mongolian medicinal preparation. Despite its long history of use for the treatment of coronary heart disease, there have been few toxicological studies of the safety profile of ZRH.

**Purpose:** In order to comprehensively elucidate the underlying mechanisms behind the observed toxicity of ZRH on rat livers in the 180-day repeated oral toxicity study, we conducted a comprehensive analysis by integrating transcriptomic and metabolomic data.

**Methods:** High-resolution mass spectrometry was conducted to evaluate the constituents of ZRH. For the acute oral toxicity study, mice were administered a dose of 32 g/(kg·d) of ZRH, while rats were instead orally administered 0.934, 1.868, or 3.736 g/(kg·d) of ZRH over a 180-day period in a 180-day repeated oral toxicity study. Conventional index and organ weights/histology were then monitored to detect any potential ZRH treatment-related toxicity. To identify key genes and metabolites involved in ZRH toxicological processes, we performed transcriptomic and metabolomic analyses of liver tissue upon ZRH treatment using RNA-seq techniques, qPCR and liquid chromatography-mass spectrometry analyses.

**Results:** A total of 60 compounds in ZRH were identified and speculated in positive and negative ion modes. Mice in the acute toxicity study exhibited no signs of ZRH-related toxicity. In a protracted oral toxicity investigation spanning 180 days, discernible elevations in liver ratios were noted in both male and female rats across all three dose cohorts, relative to the control group (*p* < 0.05 or *p* < 0.01). Upon subjecting to ZRH treatment, our transcriptomic and qPCR analyses unveiled notable upregulation of crucial genes, exemplified by *Abcb1b* and *Cyp2b2*, known for theirs involvement in liver drug transport and metabolism function. Furthermore, our untargeted metabolomic analysis provided supplementary insights, revealing significant regulation in pyrimidine metabolism, as well as alanine, aspartate, and glutamate metabolism pathways.

**Conclusion:** Our study unveils a panoramic understanding of the temporal, dosage-specific, and gene dimensions surrounding the metabolic and transcriptional shifts induced by ZRH exposure. As we peer into the future, recommendations emerge for further exploration, encompassing aspects such as time dynamics, dosage considerations, and gene-centric avenues to enhance therapeutic efficacy.

## 1 Introduction

Coronary heart disease (CHD) is a serious condition resulting from atherosclerotic coronary artery stenosis that can lead to myocardial ischemia, myocardial infarction (MI), and death (Kahleova et al., 2018; Zhou et al., 2018; Fuchs et al., 2019; Lu et al., 2020). CHD and MI are the leading causes of mortality in industrialized nations, and rates of these morbidities are steadily rising throughout the world (Sagar et al., 2012; Cao et al., 2015). While pharmaceutical agents designed to prevent such ischemia have been developed, including  calcium channel blockers, β-blockers, nitroglycerin, and angiotensin inhibitors, their efficacy is somewhat limited by their potential to induce side effects including hypotension and bradycardia (Cheung et al., 2015; Balla et al., 2018; Bosson et al., 2019; Rehman et al., 2019). Ethnic medicine practices rely on multi-component, multi-pathway approaches to treat diseases, achieve efficacious therapeutic effects through synergistic mechanisms of action (Zhang et al., 2014; Zhang et al., 2015; Xiao and Luo, 2018; Zhang et al., 2019; You et al., 2020). Ethnic medicine therapies offer a range of advantages over conventional treatments, and are widely used in China and surrounding nations for the treatment of CHD (Liu et al., 2019).

ZhuRiHeng Drop Pill (ZRH) is a traditional Mongolian medicinal preparation developed by professor Su Rongzhabu, who was awarded the title of the first national “Master of Chinese Medicine” for his outstanding achievements in the treatment of cardiovascular and cerebrovascular diseases. ZRH consists of extracts from nine Mongolian herbs, including *Choerospondiatis Fructus* (“Guangzao” in Chinese, Gz), *Myristicae Semen* (“Roudoukou” in Chinese, Rdk), *Aquilariae Lignum Resinatum* (“Chenxiang” in Chinese, Cx), *Carthami Flos* (“Honghua” in Chinese, Hh), *Tsaoko Fructus* (“Caoguo” in Chinese, Cg), *Gardeniae Fructus* (“Zhizi” in Chinese, Zz), *Ferulae Resina* (“Awei” in Chinese, Aw), *Bovis Calculus Artifactus* (“Rengong Niuhuang” in Chinese, Nh), and *Borneolum* (“Tianranbingpian” in Chinese, Bp).

ZRH treatment has been shown to be advantageous over conventional medicines when used to treat CHD patients, offering curative potential to treated individuals (Mu et al., 2019). We have previously demonstrated the ability of ZRH to improve the survival of rats suffering from acute myocardial ischemia, with such treatment being sufficient to augment cardiovascular functionality, to suppress inflammatory cytokine (TNF-α, IL-1α, and IL-1β) expression, and to inhibit ischemic myocardial damage through the regulation of mitochondrial Bax and Bcl-2 expression (Mu et al., 2019). Our previous studies on ZRH mainly focused on its role in ischaemic cardiomyopathy treatment, showing that it can effectively alleviate myocardial fibrosis, prevent cardiac remodeling and promote angiogenesis (Wang et al., 2019). ZRH was also more efficacious than individual components thereof, with these analyses having confirmed the predominant herb status of Gz and the synergistic effects of Rdk, Cg, Hh, and other herbs in this therapeutic setting (Lu et al., 2018).

During the 180-day repeated oral toxicity study, notable alterations in liver ratios were observed in both male and female rats. As such, in the present study, we integrated information from an acute, a 180-day repeated oral toxicity study, combined with metabolomic and transcriptomic analyses in order to elucidate the underlying mechanisms behind the observed toxicity of ZRH during the 180-day repeated oral toxicity study and offer a more robust foundation for the safe utilization of ZRH in the clinical treatment of patients in need.

## 2 Materials and methods

### 2.1 Chemicals and materials

*Choerospondiatis Fructus* (batch number 180601), dried and ripe fruits of *Choerospondias axillaris* (Roxb.) B. L. Burtt and A. W. Hill, *Myristicae Semen* (batch number 181001), dried semen of *Myristica fragrans* Houtt, *Aquilariae Lignum Resinatum* (batch number 181001), dried lignum of *Aquilaria sinensis* (Lour.) Spreng, *Carthami Flos* (batch number 180901), dried flowers of *Carthamus tinctorius* L., *Fructus Tsaoko* (batch number 180501), dried and ripe fruits of *Amomum tsao-ko* Crevost and Lemarié, *Gardeniae Fructus* (batch number 180501), dried and ripe fructus of *Gardenia jasminoides* J. Ellis, *Ferulae Resina* (batch number 180401), resin of *Ferula fukanensis* K. M. Shen, Borneolum (batch number 180901), extraction of branch and foliuman of *Cinnamomum camphora* (L.) J. Presl, and *Bovis Calculus Artifactus* (batch number 181001, a combination of cholic acid, tauronic acid, bilirubin, cholesterol and trace elements) were all procured from the Anguo medicine market (Baoding, China). The whole plant materials of the nine herbal materials were obtained from their original source and the botanic identification was confirmed by Professor ShengSang Na (Inner Mongolia Medical University, Hohhot, China). The specimens were deposited at the herbarium of medicinal plants (The Center for New Drug Safety Evaluation and Research, Inner Mongolia Medical University, GZ20180016, RDK20180017, CX20180018, HH20180019, CG20180020, ZZ20180021, AW20180022, NH20180023, BP20180024). The purities of all the standards were ≥98%. Thermo Fisher Scientific (MA, United States) provided methanol, acetonitrile, and formic acid (HPLC-grade).

### 2.2 Sample preparation

A mixture of *Choerospondiatis Fructus*, *Gardeniae Fructus*, and *Carthami Flos* (18:6:8) was crushed to yield a powder and then extracted twice in 70% ethanol (1:10, mass to volume ratio) under reflux conditions for 2 h each. This extract was then filtered and concentrated to prepare a final concentration equivalent to 1.02–1.03 g/mL under reduced pressure. Next, *Borneolum* and *Bovis Calculus Artifactus* (ratio 1:1) were added. A mixture of *Myristicae Semen*, *Ferulae Resina*, *Aquilariae Lignum Resinatum*, and *Fructus Tsaoko* (12:2:2:8) was ground to powder and passed through a 2 mm mesh, after which a GKSFE220-50-6L supercritical carbon dioxide instrument (Jiangsu, China) was used to extract the oil from the powder. Liquefied CO_2_ was pumped into the extraction vessel to maintain an extraction pressure of 28.5 MPa with a flow rate of 17 L/h. The extraction temperature was set to 41°C and the extraction was performed for 2 h. The full extract was then mixed with polyethylene glycol 4,000 (1:2, mass ratio) and 4% polysorbate 80 to yield the final ZRH preparation. Three batches of ZRH (20180301, 20180302, 20180303) totaling about 30 kg were prepared, of which about 20 kg was used for the acute and 180-day repeated oral toxicity study.

### 2.3 Sample solution preparation

A 10 mL volume of methanol was used to dissolve 0.5 g of ZRH powder with a magnetic stirrer (Ronghua Instrument Manufacturing Co., Ltd., Jiangsu, China), after which the solution was passed through a 0.22 µm filter to remove undissolved precipitates prior to the HPLC-Q-Exactive-MS/MS analysis of ZRH.

### 2.4 HPLC-Q-exactive-MS/MS analysis of ZRH

The identification of ingredients in ZRH was performed on a Thermo Scientific Q Exactive quadrupole-orbitrap mass spectrometer system (Thermo Fisher Scientific, United States), coupled with a Thermo Scientific Dionex Ultimate 3,000 UPLC system (Thermo Fisher Scientific, United States) equipped with a heated electrospray ionization interface (ESI). The data were captured and analyzed by Xcalibur 3.0 software (Thermo Fisher Scientific, United States). Details of the analysis had been previously published elsewhere (Liu et al., 2022).

### 2.5 Experimental animals

SPF (specific pathogen-free) KM (Kunming) mice and SD (Sprague-Dawley) rats were respectively utilized for the acute and 180-day repeated oral toxicity studies. In total, 70 KM mice (35 male, 35 female, 18–22 g) and 160 SD rats (80 male, 80 female, 5-weeks-old, males: 96.8–145.3 g, females: 102.8–145.3 g) were obtained from the Animal Experimental Center of Xinjiang (production license number: SCXK (Xin) 2016-0001, Xinjiang, China). Animals were housed in a climate-controlled facility [(25 ± 1)°C, 60%–65% relative humidity, 12 h light/dark cycle]. Males and females were housed separately in polycarbonate cages (n = 5 animals/cage). Following a 7d acclimatization period with free food and water access, animals were used for their respective toxicity studies. The Ethics Committee of Inner Mongolia Medical University approved all animal studies described herein (approval number: YKD2019146).

### 2.6 Acute oral toxicity study

**FIGURE 1:**
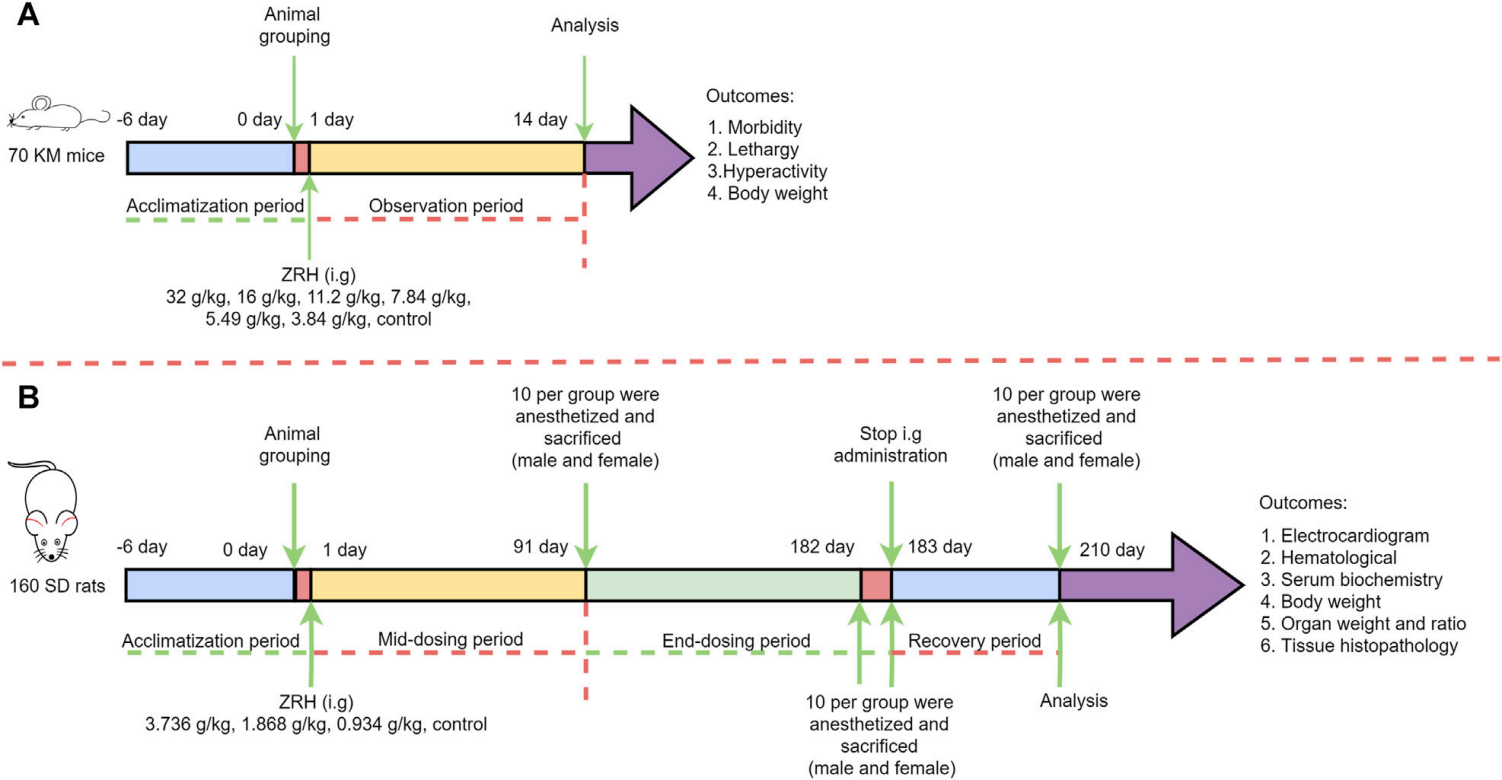
Scheme of ZRH administration and related measurement in the acute **(A)** and 180-day repeated oral toxicity **(B)** studies.

The acute oral toxicity study followed OECD guideline 425 (OECD, 2008b). Briefly, KM mice were fasted for 12 h, after which they were treated with ZRH that had been dissolved in water at a maximum concentration of 0.405 g/mL (the concentration which could pass through the mice gavage needle reluctantly). A ZRH extract dose of 16 g/(kg·d) was administered (at 08:30) by a single dose gavage in a volume of 40 mL/kg in the first preliminary experiment. The second dose was increased to 32 g/(kg·d) if one or fewer deaths occurred. That is to say, all the mice then underwent oral administration of 16 g/kg twice daily (at 08:30 and 16:00). The results in the two preliminary experiments showed that no treatment-related toxic signs or deaths were observed at both 16 g/(kg·d) and 32 g/(kg·d) dose levels over the 14-day post-treatment observation. Thus, a ZRH extract dose of 32 g/(kg·d) (685-fold higher than the dose used by human in clinical settings) was used as the highest dose in the official experiment. Thereafter, a descending sequence of dose levels was selected at about 1.43-folded intervals (16, 11.2, 7.84, 5.49, 3.84 g/kg) with a view to demonstrating any dose related toxicity and no-observed-adverse effects at the lowest dose level. All in all, 70 KM mice were randomly divided into seven groups with 10 mice in each group (five of each sex): six groups (16, 11.2, 7.84, 5.49, 3.84 and 0 g/kg) underwent oral gavage (40 mL/kg, 16 g/kg) once daily (at 08:30) while one group (32 g/kg) underwent oral gavage (40 mL/kg, 16 g/kg) twice daily (at 08:30 and 16:00). At 0.5 h post-treatment, mice were given access to food and water and monitored for acute toxicity for 24 h, after which they were monitored daily for 14 d. Body weight for these animals was measured on days 1, 2, 3, 5, 7, 10, and 14 post-dosing. After this 14-day period, mice were euthanized and a necropsy was performed (Figure 1A).

### 2.7 180-day repeated oral toxicity study

The 180-day repeated oral toxicity study was performed as per OECD guideline 452 (OECD, 2008a). The ZRH extract was freshly dissolved in sterile water before gavage (10 mL/kg), and the ZRH extract concentration was prepared as 0.3736 g/mL, which was close to 0.405 g/mL (the maximum concentration in the acute oral toxicity study) and could pass through the rat gavage needle smoothly. The highest dose level (0.3736 g/mL×10 mL/kg = 3.736 g/(kg·d)) was chosen with the aim of inducing toxic effects but not death or severe suffering. Thereafter, a descending sequence of dose levels was selected at 2-folded intervals (1.868 and 0.934 g/(kg·d)) with a view to demonstrating any dose related response and no-observed-adverse effects at the lowest dose level. In detail, a total of 160 SD rats were randomized into four groups (20 males, 20 females per group), with five rats being housed per cage and with males and females being separately housed from one another. Rats in these treatment groups were orally administered ZRH once per day at doses of 0.934, 1.868, or 3.736 g/kg via gavage, with control rats being administered an equivalent volume of sterile water. These ZRH doses were selected based upon the clinical dose. Rats had free access to food and water, and their behavior and clinical symptoms were monitored daily, while their body weight was recorded once per week (Figure 1B).

#### 2.7.1 ECG (electrocardiogram) analysis

Rats distributed across study groups were fasted overnight, anesthetized using 0.8% pentobarbital (48 mg/kg, 6 mL/kg), and fixed to an operating table in the supine position. ECG limb leads were then placed on these animals to measure the following ECG parameters: Pa (P wave height), Q (Q wave height), R (R wave height), S (S wave height), ST (ST segment), T (T wave height), Pd (P wave width), QRS (QRS complexes width), RR (RR interval), PR (PR interval), QT (QT interval), HR (Heart rate). All parameters were collected using a SP-2006 ECG instrument (Beijing Ruolong Biotechnology Co., Ltd., China).

#### 2.7.2 Hematological analysis

Blood samples from the abdominal aorta were collected into tubes coated with EDTA-K2 and were used to analyze key hematological parameters. An XT-2000iv automatic blood analyzer (Sysmex, Japan) was employed to measure the following: red blood cell count (RBC), hemoglobin level (HGB), hematocrit level (HCT), mean corpuscular volume (MCV), mean corpuscular hemoglobin (MCH), MCH concentration (MCHC), platelet count (PLT), white blood cell count (WBC), neutrophil ratio (NEUT %), lymphocyte ratio (LYM %), monocyte ratio (MONO %), eosinophil ratio (EOS %), basophil ratio (BASO %), and reticulocyte ratio (RET %). Additionally, blood samples from the abdominal aorta were collected into tubes coated with sodium citrate and analyzed with a Thrombolyzer Compact X Fully Automatic Hemagglutination Analysis (BE, Germany) to measure prothrombin time (PT), activated partial thromboplastin time (APTT), thrombin time (TT), and fibrinogen (FIB).

#### 2.7.3 Serum biochemistry analysis

Abdominal aorta blood samples were collected into untreated tubes and were used to measure key blood biochemistry parameters with a 7100 Automatic Biochemical Analyzer (Hitachi, Japan), including albumin (ALB), total bilirubin (TBIL), creatinine (CRE), urea (UREA), creatine kinase (CK), alkaline phosphatase (ALP), triglyceride (TG), total protein (TP), alanine transaminase (ALT), aspartate transaminase (AST), total cholesterol (TCHO), glucose (GLU), glutamyl transpeptidase (GGT), K^+^, Na^+^, and Cl^−^ levels.

#### 2.7.4 Organ ratio and tissue histopathology

After blood samples had been collected, animals underwent gross necropsy. The weights of major organs (heart, liver, spleen, lung, kidney, adrenal gland, thymus, brain, testis, epididymis, uterus and ovary) were quantified, with animal body weight values being used to compute organ ratios. In addition, subsamples of these major organs were collected including the brain (brain, cerebellum, brain stem), pituitary, heart, liver, spleen, lung (including the main bronchus), kidney, adrenal gland, spinal cord, thymus, pancreas, esophagus, stomach, cecum, duodenum, jejunum, ileum, colon, rectum, lymph nodes (neck, mesenteric lymph nodes), trachea, thyroid (including parathyroid glands), aorta, bladder, testes, epididymis, prostate, seminal vesicles, ovaries and fallopian tubes, uterus and cervix, vagina, salivary glands, eyes, skin, breast, bone, skeletal muscle, sciatic nerve, bone marrow (sternum). Any other tissues exhibiting evidence of gross abnormalities or masses were additionally collected. Following collection, these tissues (control and 3.736 g/kg groups) were transferred to 15% neutral-buffered formalin for fixation. Samples from the control and the 3.736 g/kg groups were then dehydrated, embedded with paraffin, sliced to generate 3 µm-thick sections, stained by using hematoxylin and eosin (H&E), and assessed via Leica DM2000 microscope (Leica Co., Germany). During these histological analyses, tissues were assessed for any evidence of necrosis, inflammatory cell infiltration, fibrosis, degeneration, or blood extravasation.

#### 2.7.5 Collection of liver tissue

After a 180-day repeated oral administration, rats were anesthetized using 0.8% pentobarbital and decapitated for liver tissue collection. These tissues were then used for transcriptome and metabolome analyses.

### 2.8 RNA sequencing

Liver tissue total RNA extraction was performed using the mirVana™ miRNA Isolation Kit from Ambion (United States), following the recommended protocol. The RNA integrity was assessed using the Bioanalyzer (Agilent 2,100, United States). Samples with a RNA Integrity Number (RIN) of ≥7 were selected for further analysis. For library construction, the TruSeq Stranded mRNA LTSample Prep Kit from Illumina (United States) was utilized, following the manufacturer’s protocol. The resulting libraries were subjected to sequencing on an Illumina HiSeqTM 2,500 platform, generating paired-end reads of 150 bp. Raw data underwent quality control and cleaning using Trimmomatic, resulting in clean reads by removing poly-N-containing and low-quality reads. Hisat2 was employed to map the clean reads to the reference genome. The read counts for each gene were obtained using htseq-count, and the fragments per kilobase of exon model per million mapped fragments (FPKM) were determined using cufflinks. To identify differentially expressed genes (DEGs), the DESeq package functions were utilized to estimate the negative binomial test and size factors. A criterion of *p*-value <0.05 and |LogFC| > 1 was employed to define significantly differentially expressed genes. To explore gene expression patterns, hierarchical cluster analysis was used on the identified DEGs. For further analysis, the KEGG pathway and Gene Ontology (GO) enrichment of DEGs were assessed using R.

### 2.9 Quantitative real-time PCR analysis

Quantitative real-time PCR analysis was carried out using SweScript All-in-One RT SuperMix for qPCR (Servicebio, G3337) and 2×Universal Blue SYBR Green qPCR Master Mix (Servicebio, G3326) using an, ETC811 Real-Time PCR system (Eastwin, Beijing). GAPDH was used as an internal reference. The primers were shown in Table 1.The relative mRNA level was calculated using the 2^−ΔΔCT^ method.

**TABLE 1:** Quantitative PCR primers.

Gene name	Genebank number	Primer sequence (5′-3′)
GAPDH	NM_017008.4	Forward: CTG​GAG​AAA​CCT​GCC​AAG​TAT​G
		Reverse: GGT​GGA​AGA​ATG​GGA​GTT​GCT
ABCB1B	NM_012623.3	Forward: GTG​TCA​CGT​GAG​GTC​GTG​AT
		Reverse: TTC​CGT​GGA​TGA​TAG​CAG​CG
CYP2B2	NM_001198676.1	Forward: CCT​CCT​AAG​TTC​ATT​CTC​CAG​CC
		Reverse: CAT​CCA​TGC​AGG​ACT​CAC​TTT​CT

### 2.10 Non-targeted metabolomics

The procedure began by adding 30 mg of the sample into a 1.5 mL Eppendorf tube containing two steel balls and L-2-chlorophenylalanine (4 μg/mL) dissolved in a methanol-water solution (400 μL, in a ratio of 4:1, v/v). These samples were then placed in a freezer at −40°C for 2 min and subsequently ground at a frequency of 60 Hz for 2 min. The entire sample was then subjected to ultrasonic extraction for 10 min while in an ice-water bath, followed by storage at −40°C for 30 min. The resulting extract was centrifuged for approximately 10 min at 4°C and 13,000 revolutions per minute. From the resulting supernatant, 300 μL was transferred to a glass vial and then freeze-dried. Next, a mixture of 300 μL of methanol and water (in a 4:1 ratio, v/v) was added to all samples, and the mixture was vortexed for 30 s. The samples were subjected to ultrasonic extraction for 3 min in an ice-water bath, followed by placement at −40°C for 2 h. Afterward, the samples were centrifuged at 12,000 rpm for 10 min at 4°C. From each tube, 150 μL of the supernatant was collected and transferred to LC vials, which were then stored at a cold temperature of −80°C until analysis by LC-MS.A liquid mass spectrometry system composed of ACQUITY UPLC I-Class in series with Q-Exactive plus quadrupole-orbitrap mass spectrometer was used to perform the metabolic profile analysis in both ESI positive and ESI negative ion modes.

The samples were separated using an ACQUITY UPLC HSS T3 column (2.1 × 100 mm, 1.8 μm) at a flow rate of 0.35 mL/min, with the column temperature set at 45°C and an injection volume of 3 μL. The elution gradient system consisted of solvent A (water containing 0.1% formic acid, v/v) and solvent B (acetonitrile containing 0.1% formic acid, v/v). The separation process followed this gradient protocol: 0–2 min 5% B∼5% B, 2–4 min 5% B∼30% B, 4–8 min 30% B∼50% B, 8–10 min 50% B∼80% B, 10–14 min 80% B-100% B, 14–15 min 100% B∼100% B, 15–15.1 min 100% B∼5% B, 15.1–16 min 5% B∼5% B. Throughout the analysis, all samples were maintained at a temperature of 4°C. The mass range for analysis was from m/z 100 to 1200. The resolution for both HCD MS/MS and MS scans was set at 70,000. Collision energy levels of 10, 20, and 40 eV were used. The mass spectrometer settings were as follows: a spray voltage of 3,800 V (positive) and 3,000 V (negative), a capillary temperature of 320°C, and an auxiliary gas heater temperature of 350°C. Quality control (QC) samples were injected regularly during the analysis to assess data repeatability and ensure the reliability of the results.

Data analysis for the LC-MS data involved several steps conducted using the Progenesis QI software (Version 2.3). The matrix was imported into R, which performed PCA to observe the samples’ overall distribution and the analysis process’s general stability. The metabolites that differ among the groups were identified by the partial least squares discriminant analysis (PLS-DA) and orthogonal partial least-squares-discrimination analysis (OPLS-DA). A 200-response permutation testing (RPT) and 7-fold cross-validation were used to assess the model quality to prevent overfitting. The OPLS-DA model’s variable importance in projection (VIP) values was considered for grading the total impact of every parameter on the group discrimination. The *t*-test was also utilized to see whether the groups’ metabolite differences were significant. The VIP values for Differential metabolites were selected based on statistical analysis (VIP ˃ 1.0 and *p*-values ˂ 0.05).

### 2.11 Statistical analysis

All analyses were presented as means and standard deviation. The organ weight, organ ratio, ECG, hematological and serum biochemistry parameters were measured for each animal in each group. Bartlett’s test for homogeneity of variance was performed first. If the variance was homogeneous, One-way ANOVA was applied and significant results were tested using LSD Test. If the variance was not homogeneous, the Tamhane’s T2 test was applied. SPSS20.0 was used for statistical analysis. Similarly, Two-way ANOVA followed by the least significant difference (LSD) were used to conduct the body weight analysis. *p*-value less than 0.05 was considered as significant.

## 3 Results

### 3.1 Characterization of chemical constituents in ZRH by high resolution mass spectrometry

**FIGURE 2:**
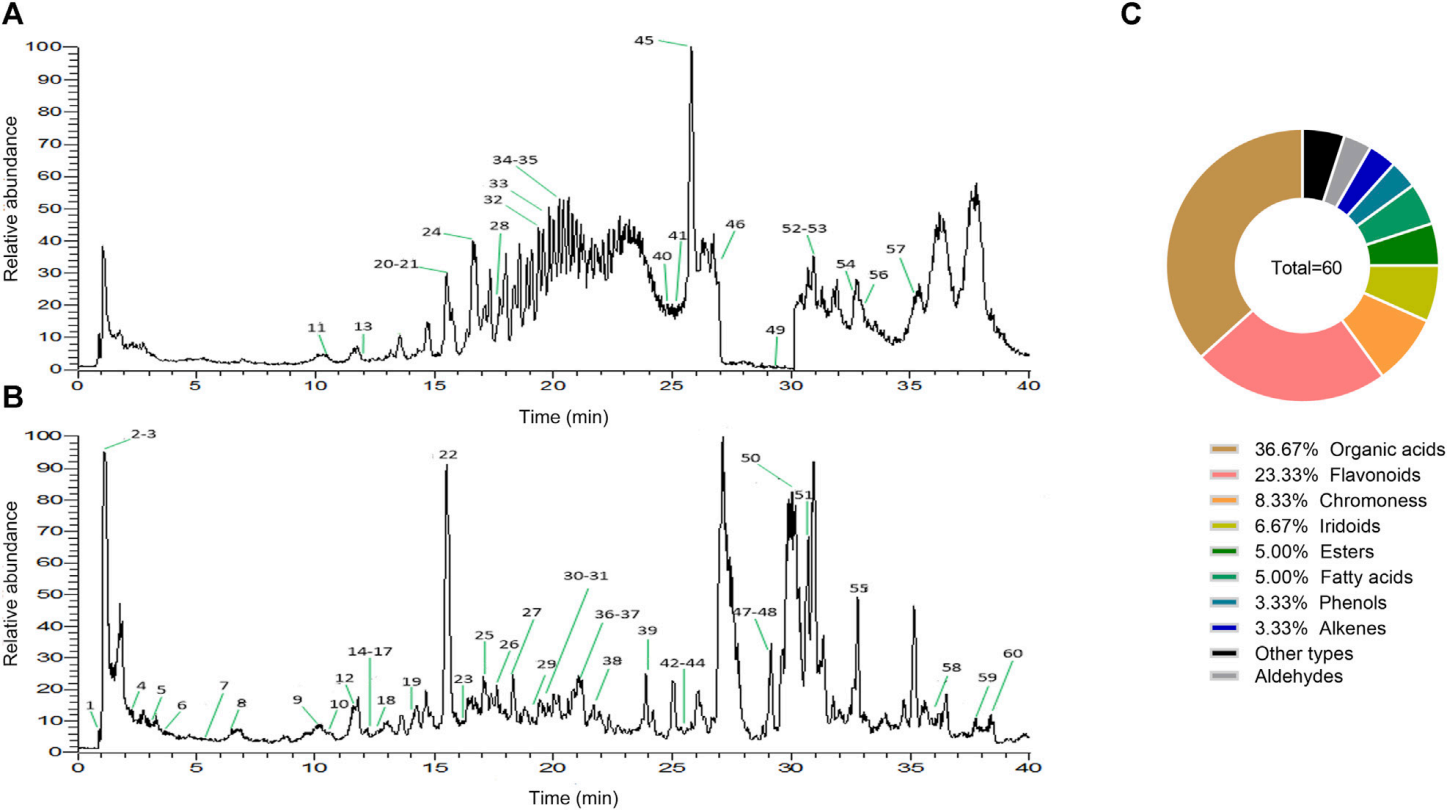
Characterization of chemical constituents in ZRH by high resolution mass spectrometry. Base peak mass spectrum of ZRH with **(A)** positive and **(B)** negative modes in the HPLC-Q-Exactive-MS/MS analysis. **(C)** ZRH Composition.

From the results of the precise mass-to-charge ratio (m/z) and secondary fragments, a total of 60 compounds in ZRH were identified and speculated in positive and negative ion modes (Figure 2), including 22 organic acids, 14 flavonoids, 5 chromoness, 4 iridoids, 3 esters, 3 fatty acids, 2 phenols, 2 alkenes, 2 aldehydes and 3 other types (Figure 2). Part of the compounds were unambiguously identified through comparisons with reference standard in terms of retention time and mass spectra (Table 2).

**TABLE 2:** Compound list for ZRH identified by HPLC-Q-Exactive-MS/MS analysis.

NO.	Compounds	t_R_/min	Formula	Ionization mode	m/z		δ/(ppm)	Fragment ions	Classification
					Theoretical	Measured			
1	Quinic acid	1.04	C_7_H_12_O_6_	[M-H] -	191.05501	191.05330	−8.974	191.05330, 173.04311, 85.02716	Organic acids
2	L(+)-Tartaric acid	1.10	C_4_H_6_O_6_	[M-H]-	149.00806	149.00670	−9.156	149.00670, 103.03772, 87.00642, 74.02245, 72.99093, 59.01182	Organic acids
3	citric acid	1.18	C_6_H_8_O_7_	[M-H] -	191.01862	191.01714	−7.795	191.01714, 173.00621, 111.00628	Organic acids
4	Monoglyceride citrate	2.16	C_9_H_14_O_9_	[M-H]-	265.05540	265.05438	−3.880	265.05438, 173.00641, 111.00632	Esters
5	Gallic acid	3.17	C_7_H_6_O_5_	[M-H]-	169.01314	169.01146	−9.998	169.01146, 125.02191, 127.02626, 97.02721	Organic acids
6	Methyll citrate	3.78	C_7_H_10_O_7_	[M-H]-	205.03427	205.03253	−8.531	205.03253, 173.00592, 143.03229, 111.00626, 87.00640	Esters
7	Gentisic	5.22	C_7_H_6_O_4_	[M-H]-	153.01823	153.01671	−9.967	153.01671, 108.04317, 91.83106	Organic acids
8	3-Phenylpropionic acid	6.40	C_9_H_10_O_2_	[M-H]-	149.05970	149.05824	−9.836	149.05824	Organic acids
9	chlorogenic acid	10.39	C_16_H_18_O_9_	[M-H] -	353.08670	353.08606	−1.837	353.08606, 191.05333	Organic acids
10	Shanzhiside	10.62	C_16_H_24_O_11_	[M-H] -	391.12348	391.12128	−5.645	391.12128, 167.06853	Iridoids
11	Hydroquinone	10.82	C_6_H_6_O_2_	[M + H]+	111.04405	111.04504	8.861	111.04504, 110.06110, 55.05545	Phenols
12	3,4-Dihydroxybenzoic acid	11.46	C_7_H_6_O_4_	[M-H]-	153.01823	153.01669	−1.545	153.01669, 109.02705, 108.04298	Organic acids
13	Syringaldehyde	11.95	C_9_H_10_O_4_	[M + H]+	183.06518	183.06668	8.165	183.06668, 155.10754, 140.08000, 123.04501, 95.05024	Aldehydes
14	Gardoside	12.12	C_16_H_22_O_10_	[M-H] -	373.11292	373.11340	1.278	373.11340, 167.03247	Iridoids
15	2-Isoprpryl malic acid	12.14	C_7_H_12_O_5_	[M-H]-	175.06009	175.05847	−9.311	175.05847, 115.03752, 113.05829, 85.06358	Organic acids
16	mussaenosidic acid	12.30	C_16_H_24_O_10_	[M-H] -	375.12857	375.12521	−8.966	375.12521, 213.07413	Organic acids
17	Deacetylasperulosidic acid	12.32	C_16_H_22_O_11_	[M-H] -	389.10783	389.10538	−6.317	389.10538, 165.05289	Iridoids
18	Procyanidin B2	12.60	C_30_H_26_O_12_	[M-H]-	577.13405	577.13843	7.585	577.13843, 179.05315	Flavonoids
19	Caffeic acid	14.16	C_9_H_8_O_4_	[M-H]-	179.03388	179.03238	−8.407	179.03238, 135.04268	Organic acids
20	phenylacetaldehyde	15.52	C_8_H_8_O	[M + H]+	121.06479	121.06580	8.330	121.06580, 91.05540, 77.03985, 65.03984	Aldehydes
21	Geniposide	15.55	C_17_H_24_O_10_	[M + Na]+	411.12726	411.12860	−5.368	411.12860	Iridoids
22	Vanillic acid	15.76	C_8_H_8_O_4_	[M-H]-	167.03388	167.03226	−1.625	167.03226, 152.00893, 108.01902	Organic acids
23	Ethyl gallate	16.10	C_9_H_10_O_5_	[M-H]-	197.04444	197.04298	−7.460	197.04298, 169.01155, 124.01389	Flavonoids
24	Agarotetrol	16.97	C_17_H_18_O_6_	[M + H]+	319.11761	319.11905	4.497	319.11905, 301.10901, 283.09833, 255.10303, 227.10776, 164,04750	Chromones
25	Hydroxysafflor yellow A	17.19	C_27_H_32_O_16_	[M-H]-	611.16066	611.15735	−5.418	611.15735, 353.06302	Flavonoids
26	Taxifolin	17.67	C_15_H_12_O_7_	[M-H]-	303.04992	303.04843	−4.947	303.04843, 125.02198	Flavonoids
27	Salicylic acid	18.47	C_7_H_6_O_3_	[M-H]-	137.02332	137.02196	−9.929	137.02196, 94.02755, 93.03232, 65.03764	Organic acids
28	Methyl cinnamate	18.51	C_10_H_10_O_2_	[M + H]+	163.07535	163.07629	5.727	131.05023, 121.10234, 107.05025, 103.05519	Esters
29	Isoquercitrin	18.84	C_21_H_20_O_12_	[M-H] -	463.08710	463.08466	−5.274	463.08466, 301.03217, 255.02670	Flavonoids
30	safflor yellow A	19.39	C_27_H_35_O_15_	[M-H]-	598.18922	598.18524	−6.656	598.18524	Flavonoids
31	Rutin	19.51	C_27_H_30_O_16_	[M-H]-	609.14501	609.14172	−5.403	609.14172, 300.02469, 271.02185	Flavonoids
32	quercetin	19.64	C_15_H_10_O_7_	[M + H]+	303.04992	303.05182	6.239	303.05182, 229.05084, 257.04620	Flavonoids
33	Ellagic acid	19.97	C_14_H_6_O_8_	[M + H]+	303.01354	303.01547	6.357	303.01547, 285.00488	Organic acids
34	(-)-Epicatechin	20.61	C_15_H_14_O_6_	[M + H]+	291.08631	291.08801	5.824	291.08801, 289.07269, 165.05598, 139.04007	Flavonoids
35	Coumarin	20.68	C_9_H_6_O_2_	[M + H]+	147.04405	147.04503	6.624	147.04503, 119.05021, 103.04573, 95.05064, 91.05537	Coumarins
36	Nicotiflorin	20.69	C_27_H_30_O_15_	[M-H] -	593.15009	593.14716	−4.951	593.14716, 325.06290	Flavonoids
37	Kaempferol 7-O-glucoside	20.93	C_21_H_20_O_11_	[M-H]-	447.09218	447.09146	−1.628	447.09146, 284.03000	Flavonoids
38	Methylellagic acid	21.79	C_15_H_8_O_8_	[M-H]-	315.01354	315.01199	−4.932	315.01199, 300.99188	Organic acids
39	Naringetol	23.73	C_15_H_12_O_5_	[M-H]-	271.06009	271.05881	−1.290	271.05881, 151.00104, 120.05093, 119.04774, 107.01142	Flavonoids
40	D(+)-Camphor	24.88	C_10_H_16_O	[M + H]+	153.12739	153.12837	6.389	153.12837, 108.08988, 95.08677, 81.07110, 69.07112, 55.01922	Ketones
41	6-Hydroxy-2-(2-phenylethyl)chromone	25.25	C_17_H_14_O_3_	[M + H]+	267.10157	267.10294	5.126	267.10294, 176.04791, 161.13408, 137.09688	Chromones
42	apigenin	25.43	C_15_H_10_O_5_	[M-H]-	269.04444	269.04306	−5.166	269.04306	Flavonoids
43	dehydrocholic acid	25.54	C_24_H_34_O_5_	[M-H]-	401.23225	401.23148	−1.921	401.23148	Organic acids
44	Kaempferol	25.57	C_15_H_10_O_6_	[M-H]-	285.03936	285.03790	−5.138	285.03790	Flavonoids
45	5,8-dihydroxy-2-(2-phenylethyl)chromone	25.69	C_17_H_14_O_4_	[M + H]+	283.09648	283.09808	5.633	283.09808, 192.04300	Chromones
46	Myrcene	26.98	C_10_H_16_	[M + H]+	137.13247	137.13342	6.876	137.13342, 93.07101, 69.07106, 79.05540	Alkenes
47	stearic acid	28.96	C_18_H_36_O_2_	[M-H]-	283.26315	283.26245	−2.495	283.26245	Organic acids
48	Glycocholic acid	29.18	C_26_H_43_NO_6_	[M-H]-	464.30066	464.29800	−5.739	464.29800, 74.02259	Organic acids
49	6-methoxy-2-(2-phenylethyl)chromen-4-one	29.23	C_18_H_16_O_3_	[M + H]+	281.11722	281.11865	5.084	281.11865, 190.06349, 151.03923	Chromones
50	Deoxycholic acid	30.63	C_24_H_40_O_4_	[M-H]-	391.28428	391.28229	−5.102	391.28229	Organic acids
51	Hyocholic acid	30.85	C_24_H_40_O_5_	[M-H]-	407.27920	407.27710	−5.158	407.27710	Organic acids
52	2,6-Dimethoxyphenol	31.21	C_8_H_10_O_3_	[M + H]+	155.07027	155.07137	7.089	155.07137, 154.96363, 139.04002, 111.04516, 96.05373, 93.03458	Phenols
53	2-(2-phenylethyl)chromon	31.70	C_17_H_14_O_2_	[M + H]+	251.10665	251.10797	5.232	251.10797, 173.13361, 161.13316, 121.08686	Chromones
54	γ-Linolenic acid	32.43	C_18_H_30_O_2_	[M + H]+	279.23185	279.23428	8.678	279.23428, 262.22650, 261.22302, 243.21196, 195.13866	Fatty acids
55	Chenodeoxycholic acid	32.78	C_24_H_40_O_4_	[M-H]-	391.28428	391.28214	−5.485	391.28214	Organic acids
56	beta-elemene	33.01	C_15_H_24_	[M + H]+	205.19507	205.19635	6.202	205.19635, 81.07110, 67.05546, 55.05541	Alkenes
57	Elaidic acid	35.24	C_18_H_34_O_2_	[M + H]+	283.26315	283.26498	6.436	283.26498, 109.10230, 95.08684, 81.07112, 69.07116, 57.07118	Fatty acids
58	Oleanolic acid	35.91	C_30_H_48_O_3_	[M-H]-	455.35197	455.34949	−5.450	455.34949	Triterpenoids
59	palmitic acid	37.63	C_16_H_32_O_2_	[M-H]-	255.23185	255.23038	−5.786	255.23038	Fatty acids
60	Icosanoic acid	38.43	C_20_H_40_O_2_	[M-H]-	311.29445	311.29291	−4.969	311.29291, 119.14525	Organic acids

### 3.2 Effects of acute oral toxicity study with ZRH on behavioral changes and body weight in mice

**FIGURE 3:**
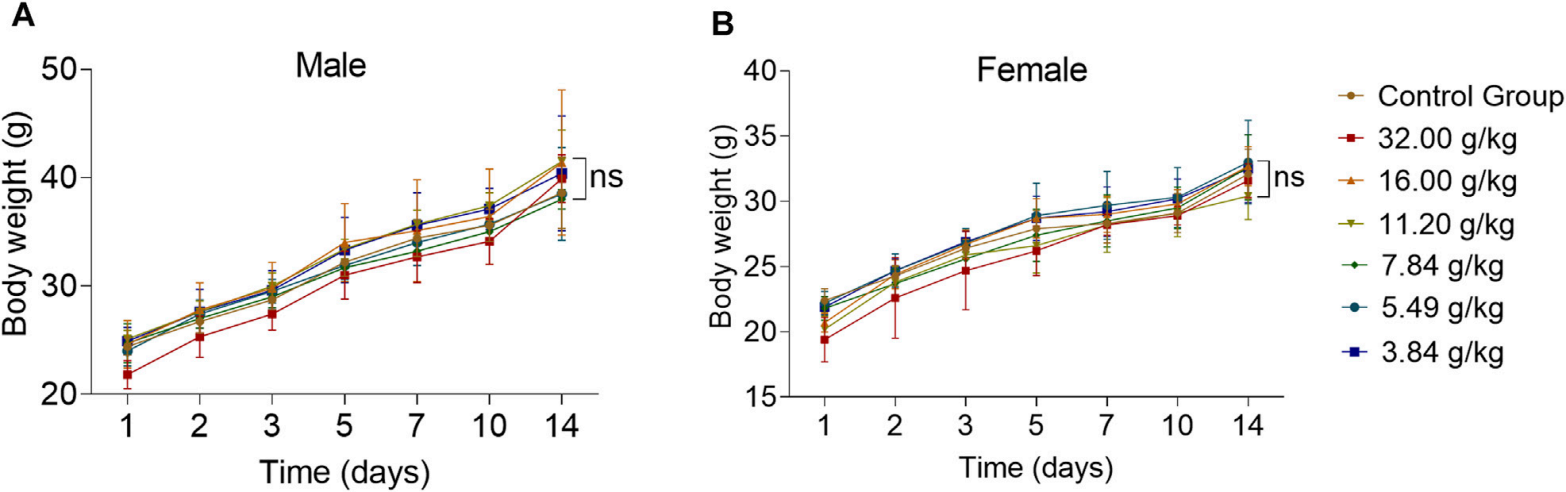
Effects of acute oral toxicity study with ZRH on behavioral changes and body weight in mice. Bodyweight of male **(A)** and female **(B)** KM mice during the acute toxicity study, all results are expressed as the mean ± SD with one-way ANOVA followed by the LSD multiple comparisons test, *n* = 5.

For an acute toxicity study, KM mice were orally administered individual ZRH doses of 0, 3.84, 5.49, 7.84, 11.20, 16.00, and 32.00 g/kg, and were then monitored for 14 d thereafter. We observed no evidence of behavioral changes (morbidity, lethargy, hyperactivity) or body weight attributable to treatment in any of these animals over this period (Figure 3) (*p* > 0.05).

### 3.3 180-day repeated oral toxicity study

#### 3.3.1 Effects of 180 days of oral treatment with ZRH on body weight of rats

**FIGURE 4:**
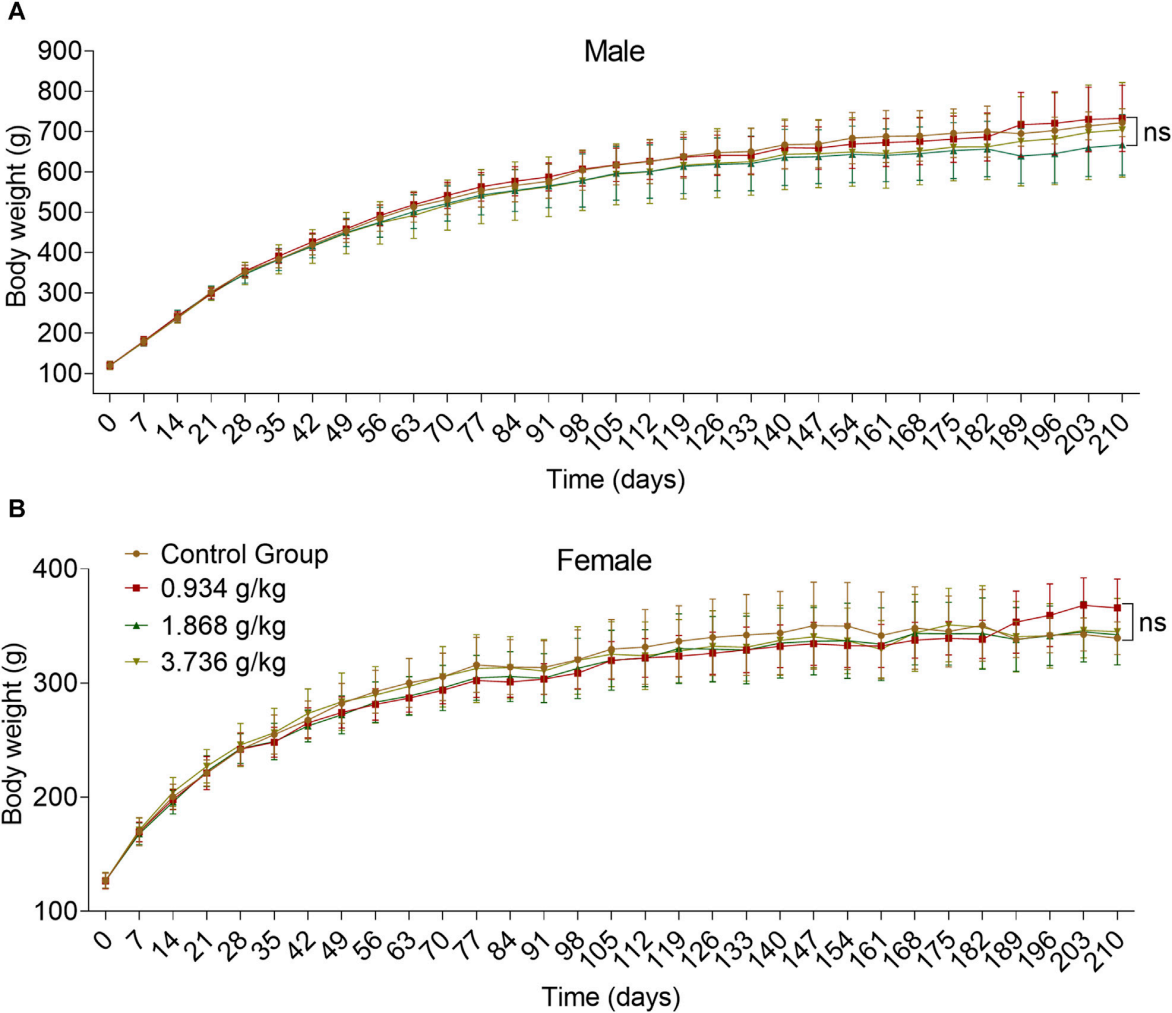
Effects of 180 days of oral treatment with ZRH on body weight of rats. Bodyweight of male **(A)** and female **(B)** SD rats during the 180-day repeated oral toxicity study, all results are expressed as the mean ± SD with one-way ANOVA followed by the LSD multiple comparisons test, D91 *n* = 5, D183/D183 *n* = 10, D210 *n* = 5.

There were no significant differences in body weight when comparing sex-matched control and treatment groups over the study period, with overall upward trends being observed over time (Figure 4) (*p* > 0.05).

#### 3.3.2 Effects of 180 days of oral treatment with ZRH on ECG parameters of rats

Male rats did not exhibit any significant changes in ECG parameters at the measured time points (Supplementary Table S1). When ECG parameters for female rats were analyzed (Supplementary Table S2), we found Pd to be significantly increased at the mid-dosing period in the 1.868 g/kg group (*p* < 0.01), while RR was increased at the end-dosing (*p* < 0.01) and recovery period (*p* < 0.05) in the 1.868 g/kg group as compared to control rats. In addition, HR was significantly reduced in the 1.868 g/kg group rats at the end-dosing period (*p* < 0.01), while both QRS and HR were reduced at the recovery period in this 1.868 g/kg group (*p* < 0.05), and QRS was also altered at the end of recovery period in the 0.934 g/kg group (*p* < 0.01) as compared to control rats.

#### 3.3.3 Effects of 180 days of oral treatment with ZRH on hematological parameters of rats

Hematological analyses of male rats (Supplementary Table S3) revealed a significant increase in EOS % at the 1.868 g/kg (*p* < 0.05) at the end of mid-dosing period and in both HCT and HGB at a 3.736 g/kg dose (*p* < 0.01) at the end-dosing period as compared to controls. Furthermore, at the mid-dosing time point period, MCHC was significantly decreased in the 1.868 g/kg group (*p* < 0.01). No significant differences in hematological parameters were observed in female rats (Supplementary Table S4), and coagulation parameters were unaffected in rats of either sex during the study (Supplementaryable S3, S4).

#### 3.3.4 Effects of 180 days of oral treatment with ZRH on serum biochemistry parameters of rats

Serum biochemistry analyses of male rats (Supplementary Table S5) revealed significant increases in TG levels at the 0.934 g/kg dose (*p* < 0.05) at the end of mid-dosing period, while at the end-dosing period, the K^+^ levels in these rats were elevated at doses of 0.934 g/kg (*p* < 0.05) and 3.736 g/kg (*p* < 0.001) relative to the control rats. These results further revealed that GLU levels were significantly reduced in rats in the 3.736 g/kg group at the end-dosing period relative to control rats (*p* < 0.05). Serum biochemistry analyses of female rats (Supplementary Table S6) revealed UREA levels to be significantly elevated in all three treatment groups at the end-dosing period (*p* < 0.01 or *p* < 0.001), while GGT levels were elevated at doses of 1.868 g/kg (*p* < 0.05) and 3.736 g/kg (*p* < 0.01) at the end-dosing period, and K^+^ levels were elevated at doses of 1.868 g/kg (*p* < 0.05) and 3.736 g/kg (*p* < 0.01) at the end of mid-dosing period, and at a dose of 3.736 g/kg (*p* < 0.001) at the end-dosing period as compared to controls. These analyses further revealed TP levels to be significantly reduced at a dose of 3.736 g/kg (*p* < 0.01) at the end-dosing period as compared to control animals. Collectively, only changes in serum UREA levels in female rats were found following treatment as compared to control rats.

#### 3.3.5 Effects of 180 days of oral treatment with ZRH on organ weight of rats

As was shown in Table 3, a significant increase in the absolute kidney weight of male rats in the 1.868 g/kg treatment group was observed after end-dosing period (*p* < 0.01), and a similar increase was also observed in the 0.934 g/kg treatment group at the recovery period (*p* < 0.05). Following the end-dosing period, relative kidney weight ratio values were significantly increased in all three dose groups (*p* < 0.05, *p* < 0.01 or *p* < 0.001), while at this same time point the relative adrenal gland weight ratio rose significantly at a 3.736 g/kg dose (*p* < 0.01), the relative testis weight ratio increased significantly at 0.934 g/kg (*p* < 0.05) and 1.868 g/kg (*p* < 0.05) doses, and the relative liver weight ratio increases significantly in all three treatment groups relative to controls (*p* < 0.05 or *p* < 0.01). The absolute adrenal gland weight in female rats declined significantly at a 1.868 g/kg dose at the end of the recovery period (Table 4; *p* < 0.05), while relative liver weight ratio values significantly increased at 1.868 g/kg (*p* < 0.05) and 3.736 g/kg (*p* < 0.01) doses at the end of mid-dosing period, and relative liver weight ratio values significantly rose at all three tested doses at the end-dosing period relative to control rats (*p* < 0.05 or *p* < 0.01). Overall, when relative organ weight values were analyzed, we detected significant changes in the relative liver weight ratios in both male and female rats following treatment and in the kindey weight ratios of male rats at the end of dosing as compared to control rats.

**TABLE 3:** Absolute and relative organ weight of male rats in the 180-day repeated oral toxicity.

Time point	Parameters	Groups			
		Control	0.934 g/kg	1.868 g/kg	3.736 g/kg
D91 (mid-dosing period)	Fasting weight (g)	554 ± 34	562 ± 28	556 ± 34	539 ± 82
	Heart (g)	1.52 ± 0.15	1.59 ± 0.12	1.49 ± 0.05	1.48 ± 0.19
	Liver (g)	14.7 ± 1.6	16.1 ± 2.0	16.2 ± 1.6	16.0 ± 3.0
	Spleen (g)	0.874 ± 0.094	0.900 ± 0.172	0.935 ± 0.157	0.920 ± 0.182
	Lung (g)	2.07 ± 0.25	2.10 ± 0.14	2.20 ± 0.26	2.04 ± 0.17
	Kidney(g)	3.31 ± 0.23	3.39 ± 0.30	3.65 ± 0.49	3.67 ± 0.56
	Adrenal gland (g)	0.066 ± 0.005	0.071 ± 0.011	0.067 ± 0.006	0.072 ± 0.007
	Thymus (g)	0.403 ± 0.068	0.411 ± 0.105	0.398 ± 0.096	0.462 ± 0.107
	Brain (g)	2.02 ± 0.17	2.03 ± 0.06	2.13 ± 0.13	2.09 ± 0.12
	Testis (g)	3.67 ± 0.25	3.62 ± 0.32	4.02 ± 0.64	3.62 ± 0.07
	Epididymis (g)	1.52 ± 0.14	1.56 ± 0.10	1.56 ± 0.13	1.66 ± 0.05
	Heart ratio (%)	0.274 ± 0.027	0.283 ± 0.020	0.269 ± 0.021	0.275 ± 0.010
	Liver ratio (%)	2.66 ± 0.23	2.86 ± 0.30	2.90 ± 0.20	2.96 ± 0.15
	Spleen ratio (%)	0.158 ± 0.018	0.160 ± 0.029	0.168 ± 0.026	0.172 ± 0.035
	Lung ratio (%)	0.375 ± 0.043	0.375 ± 0.026	0.397 ± 0.054	0.382 ± 0.034
	Kidney ratio (%)	0.598 ± 0.029	0.603 ± 0.032	0.658 ± 0.082	0.682 ± 0.067
	Adrenal gland ratio (%)	0.012 ± 0.001	0.013 ± 0.002	0.012 ± 0.002	0.013 ± 0.002
	Thymus ratio (%)	0.073 ± 0.015	0.073 ± 0.018	0.071 ± 0.015	0.087 ± 0.027
	Brain ratio (%)	0.366 ± 0.040	0.362 ± 0.017	0.383 ± 0.020	0.396 ± 0.073
	Testis ratio (%)	0.665 ± 0.065	0.648 ± 0.086	0.726 ± 0.119	0.683 ± 0.091
	Epididymis ratio (%)	0.276 ± 0.035	0.279 ± 0.023	0.281 ± 0.017	0.315 ± 0.052
D183 (end-dosing period)	Fasting weight (g)	693 ± 74	661 ± 50	648 ± 69	637 ± 63
	Heart (g)	1.73 ± 0.23	1.65 ± 0.23	1.73 ± 0.15	1.73 ± 0.25
	Liver (g)	16.4 ± 2.4	17.2 ± 2.1	16.6 ± 1.9	17.9 ± 2.5
	Spleen (g)	0.985 ± 0.193	1.044 ± 0.179	1.001 ± 0.176	1.049 ± 0.267
	Lung (g)	1.98 ± 0.21	2.09 ± 0.24	2.09 ± 0.25	2.06 ± 0.21
	Kidney(g)	3.38 ± 0.28	3.58 ± 0.25	3.76 ± 0.19^**^	3.59 ± 0.32
	Adrenal gland (g)	0.057 ± 0.007	0.056 ± 0.006	0.058 ± 0.005	0.063 ± 0.010
	Thymus (g)	0.329 ± 0.094	0.315 ± 0.092	0.271 ± 0.058	0.300 ± 0.042
	Brain (g)	2.24 ± 0.10	2.22 ± 0.09	2.22 ± 0.15	2.18 ± 0.14
	Testis (g)	3.47 ± 0.81	3.90 ± 0.32	3.78 ± 0.30	3.56 ± 0.39
	Epididymis (g)	1.583 ± 0.252	1.660 ± 0.206	1.643 ± 0.199	1.554 ± 0.276
	Heart ratio (%)	0.250 ± 0.023	0.248 ± 0.019	0.269 ± 0.027	0.271 ± 0.026
	Liver ratio (%)	2.36 ± 0.17	2.59 ± 0.16^*^	2.57 ± 0.11^*^	2.81 ± 0.28^**^
	Spleen ratio (%)	0.142 ± 0.020	0.158 ± 0.024	0.154 ± 0.017	0.164 ± 0.037
	Lung ratio (%)	0.287 ± 0.028	0.317 ± 0.044	0.324 ± 0.043	0.324 ± 0.024
	Kidney ratio (%)	0.489 ± 0.033	0.544 ± 0.047^*^	0.585 ± 0.056^***^	0.564 ± 0.040^**^
	Adrenal gland ratio (%)	0.008 ± 0.001	0.008 ± 0.001	0.009 ± 0.001	0.010 ± 0.002^**^
	Thymus ratio (%)	0.047 ± 0.010	0.047 ± 0.012	0.042 ± 0.007	0.047 ± 0.005
	Brain ratio (%)	0.325 ± 0.029	0.336 ± 0.020	0.345 ± 0.033	0.343 ± 0.017
	Testis ratio (%)	0.502 ± 0.114	0.593 ± 0.070^*^	0.586 ± 0.050^*^	0.561 ± 0.058
	Epididymis ratio (%)	0.230 ± 0.042	0.253 ± 0.039	0.255 ± 0.028	0.245 ± 0.042
D210 (recovery period)	Fasting weight (g)	710 ± 38	722 ± 82	658 ± 73	691 ± 116
	Heart (g)	1.81 ± 0.06	1.83 ± 0.25	1.75 ± 0.25	1.73 ± 0.29
	Liver (g)	16.4 ± 1.3	18.3 ± 3.7	16.2 ± 2.7	18.5 ± 4.4
	Spleen (g)	0.869 ± 0.078	0.972 ± 0.073	0.970 ± 0.089	0.940 ± 0.208
	Lung (g)	2.29 ± 0.51	2.24 ± 0.28	2.16 ± 0.24	2.12 ± 0.60
	Kidney(g)	3.37 ± 0.09	3.77 ± 0.26^*^	3.21 ± 0.32	3.43 ± 0.30
	Adrenal gland (g)	0.052 ± 0.008	0.060 ± 0.009	0.054 ± 0.010	0.058 ± 0.008
	Thymus (g)	0.267 ± 0.073	0.357 ± 0.118	0.333 ± 0.100	0.355 ± 0.137
	Brain (g)	2.25 ± 0.09	2.20 ± 0.08	2.11 ± 0.05	2.21 ± 0.08
	Testis (g)	3.97 ± 0.30	3.66 ± 0.12	3.96 ± 0.22	3.63 ± 0.37
	Epididymis (g)	1.63 ± 0.07	1.65 ± 0.14	1.72 ± 0.10	1.54 ± 0.11
	Heart ratio (%)	0.256 ± 0.012	0.253 ± 0.027	0.267 ± 0.029	0.251 ± 0.024
	Liver ratio (%)	2.32 ± 0.11	2.52 ± 0.25	2.45 ± 0.18	2.66 ± 0.23
	Spleen ratio (%)	0.123 ± 0.017	0.136 ± 0.014	0.149 ± 0.017	0.136 ± 0.017
	Lung ratio (%)	0.322 ± 0.068	0.311 ± 0.015	0.330 ± 0.043	0.304 ± 0.044
	Kidney ratio (%)	0.475 ± 0.019	0.525 ± 0.041	0.493 ± 0.067	0.505 ± 0.069
	Adrenal gland ratio (%)	0.007 ± 0.001	0.008 ± 0.001	0.008 ± 0.001	0.008 ± 0.000
	Thymus ratio (%)	0.038 ± 0.011	0.050 ± 0.017	0.050 ± 0.012	0.050 ± 0.012
	Brain ratio (%)	0.318 ± 0.022	0.309 ± 0.044	0.326 ± 0.048	0.327 ± 0.050
	Testis ratio (%)	0.560 ± 0.027	0.512 ± 0.054	0.608 ± 0.077	0.534 ± 0.082
	Epididymis ratio (%)	0.230 ± 0.020	0.230 ± 0.030	0.264 ± 0.030	0.227 ± 0.031

Data are expressed as mean ± SD, with one-way ANOVA, followed by the LSD, multiple comparisons test, statistically significant compared to control (^*^ *p* < 0.05; ^**^ *p* < 0.01; ^***^ *p* < 0.001; D91 *n* = 5, D183 *n* = 10, D210 *n* = 5).

**TABLE 4:** Absolute and relative organ weight of female rats in the 180-day repeated oral toxicity.

Time point	Parameters	Groups			
		Control	0.934 g/kg	1.868 g/kg	3.736 g/kg
D91 (mid-dosing period)	Fasting weight (g)	301.26 ± 18.68	291.96 ± 13.38	292.16 ± 22.51	295.64 ± 26.49
	Heart (g)	1.027 ± 0.137	1.000 ± 0.214	1.033 ± 0.163	1.001 ± 0.147
	Liver (g)	7.99 ± 0.41	8.26 ± 0.60	8.79 ± 0.95	9.58 ± 1.42
	Spleen (g)	0.595 ± 0.072	0.543 ± 0.101	0.651 ± 0.123	0.590 ± 0.146
	Lung (g)	1.57 ± 0.12	1.48 ± 0.06	1.58 ± 0.12	1.58 ± 0.25
	Kidney (g)	1.91 ± 0.25	1.90 ± 0.08	1.91 ± 0.25	1.97 ± 0.33
	Adrenal gland (g)	0.080 ± 0.011	0.073 ± 0.010	0.078 ± 0.026	0.071 ± 0.006
	Thymus (g)	0.329 ± 0.078	0.314 ± 0.085	0.319 ± 0.086	0.306 ± 0.074
	Brain (g)	1.82 ± 0.08	1.88 ± 0.15	1.74 ± 0.14	1.83 ± 0.12
	Ovary (g)	0.176 ± 0.056	0.167 ± 0.056	0.158 ± 0.034	0.154 ± 0.033
	Uterus (g)	0.860 ± 0.253	1.146 ± 0.322	1.168 ± 0.530	0.966 ± 0.257
	Heart ratio (%)	0.343 ± 0.059	0.342 ± 0.070	0.354 ± 0.057	0.338 ± 0.035
	Liver ratio (%)	2.66 ± 0.21	2.83 ± 0.18	3.00 ± 0.12^*^	3.24 ± 0.33^**^
	Spleen ratio (%)	0.197 ± 0.018	0.187 ± 0.037	0.222 ± 0.031	0.199 ± 0.044
	Lung ratio (%)	0.523 ± 0.019	0.508 ± 0.020	0.540 ± 0.024	0.534 ± 0.063
	Kidney ratio (%)	0.634 ± 0.064	0.650 ± 0.028	0.652 ± 0.045	0.664 ± 0.080
	Adrenal gland ratio (%)	0.027 ± 0.004	0.025 ± 0.003	0.027 ± 0.007	0.024 ± 0.004
	Thymus ratio (%)	0.109 ± 0.024	0.107 ± 0.028	0.108 ± 0.024	0.103 ± 0.018
	Brain ratio (%)	0.606 ± 0.018	0.643 ± 0.028	0.597 ± 0.056	0.622 ± 0.067
	Ovary ratio (%)	0.058 ± 0.016	0.057 ± 0.019	0.054 ± 0.011	0.052 ± 0.011
	Uterus ratio (%)	0.284 ± 0.072	0.391 ± 0.101	0.406 ± 0.197	0.333 ± 0.111
D182 (end-dosing period)	Fasting weight (g)	347 ± 33	335 ± 20	339 ± 31	340 ± 34
	Heart (g)	1.081 ± 0.077	1.004 ± 0.127	1.007 ± 0.136	1.044 ± 0.142
	Liver (g)	8.67 ± 0.76	9.37 ± 0.95	9.28 ± 1.21	9.54 ± 0.71
	Spleen (g)	0.647 ± 0.078	0.648 ± 0.085	0.650 ± 0.100	0.720 ± 0.074
	Lung (g)	1.44 ± 0.15	1.50 ± 0.18	1.50 ± 0.24	1.54 ± 0.23
	Kidney (g)	1.99 ± 0.14	1.96 ± 0.18	1.90 ± 0.26	2.02 ± 0.20
	Adrenal gland (g)	0.060 ± 0.016	0.062 ± 0.012	0.054 ± 0.012	0.069 ± 0.014
	Thymus (g)	0.266 ± 0.100	0.220 ± 0.032	0.238 ± 0.066	0.222 ± 0.059
	Brain (g)	1.98 ± 0.08	1.99 ± 0.13	1.91 ± 0.14	2.00 ± 0.07
	Ovary (g)	0.132 ± 0.032	0.135 ± 0.016	0.129 ± 0.034	0.151 ± 0.038
	Uterus (g)	1.418 ± 0.478	1.238 ± 0.280	1.379 ± 0.283	1.411 ± 0.544
	Heart ratio (%)	0.312 ± 0.018	0.301 ± 0.041	0.297 ± 0.022	0.306 ± 0.023
	Liver ratio (%)	2.51 ± 0.20	2.80 ± 0.28^**^	2.73 ± 0.21^*^	2.81 ± 0.21^**^
	Spleen ratio (%)	0.188 ± 0.031	0.194 ± 0.026	0.192 ± 0.028	0.213 ± 0.025
	Lung ratio (%)	0.416 ± 0.041	0.451 ± 0.056	0.442 ± 0.061	0.453 ± 0.048
	Kidney ratio (%)	0.578 ± 0.053	0.586 ± 0.062	0.561 ± 0.044	0.596 ± 0.056
	Adrenal gland ratio (%)	0.017 ± 0.005	0.019 ± 0.004	0.016 ± 0.003	0.021 ± 0.004
	Thymus ratio (%)	0.076 ± 0.024	0.066 ± 0.009	0.070 ± 0.018	0.065 ± 0.015
	Brain ratio (%)	0.575 ± 0.044	0.596 ± 0.042	0.565 ± 0.036	0.592 ± 0.051
	Ovary ratio (%)	0.038 ± 0.009	0.040 ± 0.005	0.038 ± 0.008	0.044 ± 0.010
	Uterus ratio (%)	0.416 ± 0.161	0.374 ± 0.097	0.405 ± 0.065	0.425 ± 0.191
D210 (recovery period)	Fasting weight (g)	354 ± 22	331 ± 16	332 ± 28	335 ± 23
	Heart (g)	1.014 ± 0.096	1.118 ± 0.138	1.028 ± 0.118	1.126 ± 0.078
	Liver (g)	8.01 ± 0.55	8.84 ± 0.42	8.32 ± 0.66	8.91 ± 1.23
	Spleen (g)	0.582 ± 0.064	0.626 ± 0.061	0.610 ± 0.086	0.616 ± 0.105
	Lung (g)	1.51 ± 0.16	1.50 ± 0.13	1.52 ± 0.18	1.47 ± 0.11
	Kidney (g)	1.87 ± 0.22	1.95 ± 0.23	1.83 ± 0.20	1.89 ± 0.18
	Adrenal gland (g)	0.073 ± 0.007	0.064 ± 0.010	0.054 ± 0.017^*^	0.077 ± 0.014
	Thymus (g)	0.227 ± 0.084	0.257 ± 0.061	0.199 ± 0.052	0.175 ± 0.018
	Brain (g)	2.00 ± 0.13	1.93 ± 0.07	1.98 ± 0.05	1.99 ± 0.16
	Ovary (g)	0.128 ± 0.020	0.170 ± 0.045	0.129 ± 0.029	0.151 ± 0.016
	Uterus (g)	1.414 ± 0.420	1.091 ± 0.153	1.962 ± 1.084	1.550 ± 0.578
	Heart ratio (%)	0.288 ± 0.036	0.338 ± 0.044	0.309 ± 0.011	0.337 ± 0.028
	Liver ratio (%)	2.28 ± 0.27	2.68 ± 0.23	2.51 ± 0.19	2.66 ± 0.36
	Spleen ratio (%)	0.165 ± 0.019	0.190 ± 0.025	0.184 ± 0.028	0.183 ± 0.024
	Lung ratio (%)	0.428 ± 0.065	0.454 ± 0.058	0.458 ± 0.036	0.440 ± 0.053
	Kidney ratio (%)	0.527 ± 0.042	0.590 ± 0.081	0.551 ± 0.037	0.565 ± 0.036
	Adrenal gland ratio (%)	0.021 ± 0.001	0.019 ± 0.003	0.017 ± 0.006	0.023 ± 0.005
	Thymus ratio (%)	0.065 ± 0.028	0.078 ± 0.019	0.059 ± 0.013	0.053 ± 0.009
	Brain ratio (%)	0.566 ± 0.044	0.586 ± 0.043	0.601 ± 0.059	0.595 ± 0.064
	Ovary ratio (%)	0.036 ± 0.005	0.052 ± 0.014	0.039 ± 0.006	0.045 ± 0.006
	Uterus ratio (%)	0.399 ± 0.116	0.330 ± 0.044	0.592 ± 0.310	0.462 ± 0.173

Data are expressed as mean ± SD, with one-way ANOVA, followed by the LSD, multiple comparisons test, statistically significant compared to control (^*^ *p* < 0.05; ^**^ *p* < 0.01; ***p < 0.001; D91 *n* = 5, D182 *n* = 10, D210 *n* = 5).

#### 3.3.6 Effects of 180 days of oral treatment with ZRH on histopathologic of rats

**FIGURE 5:**
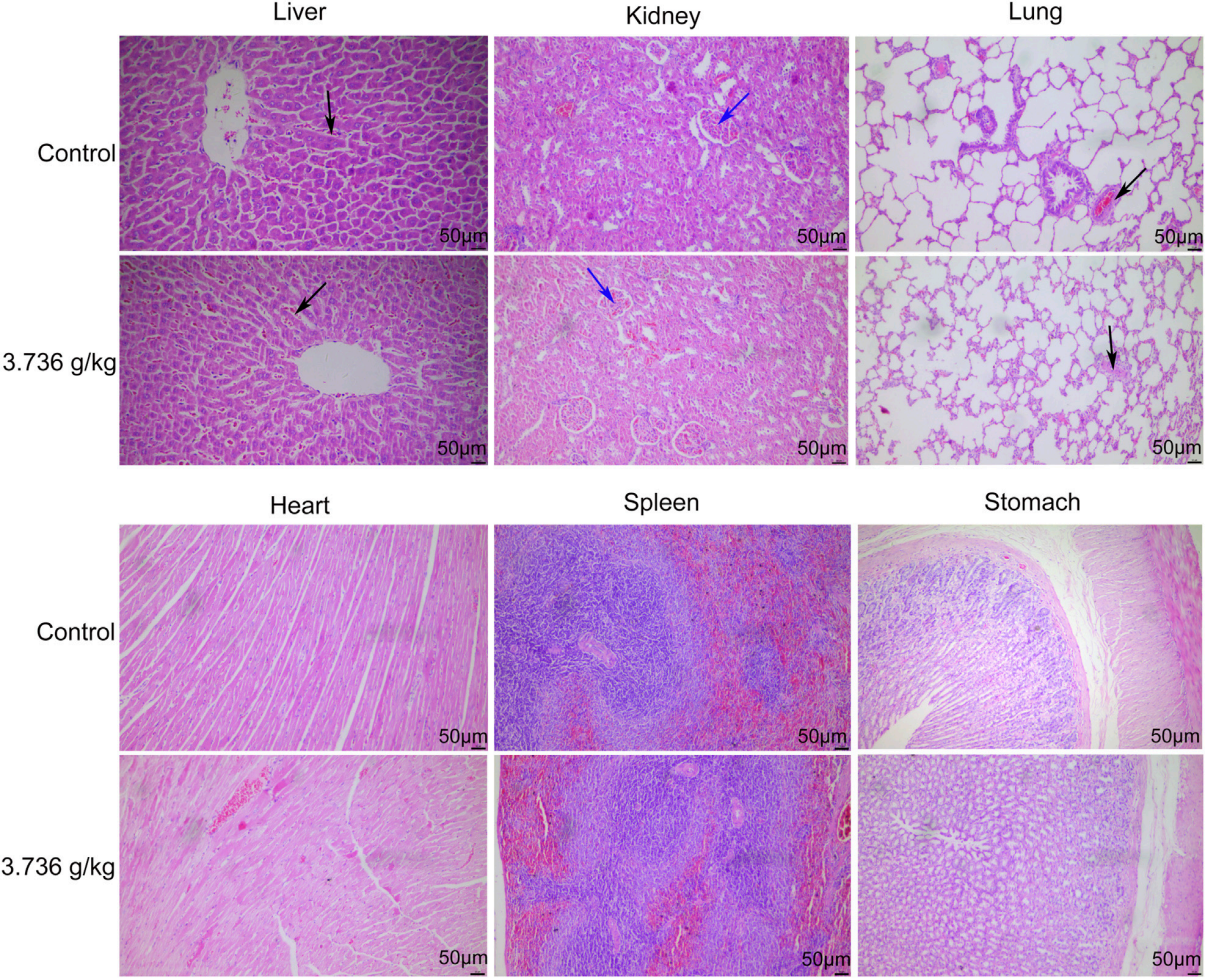
Effects of 180 days of oral treatment with ZRH on histopathological parameters of male rats. H&E staining (×20) of the liver of control and 3.736 g/kg; kidney of control and 3.736 g/kg; lung of control and 3.736 g/kg; heart of control and 3.736 g/kg; spleen of control and 3.736 g/kg; stomach of control and 3.736 g/kg of the male rats, scale bar = 50 μm. The black arrow indicates spontaneous inflammatory cell infiltration including lung/liver, while the blue arrow indicates calcium deposition in renal tubules.

**FIGURE 6:**
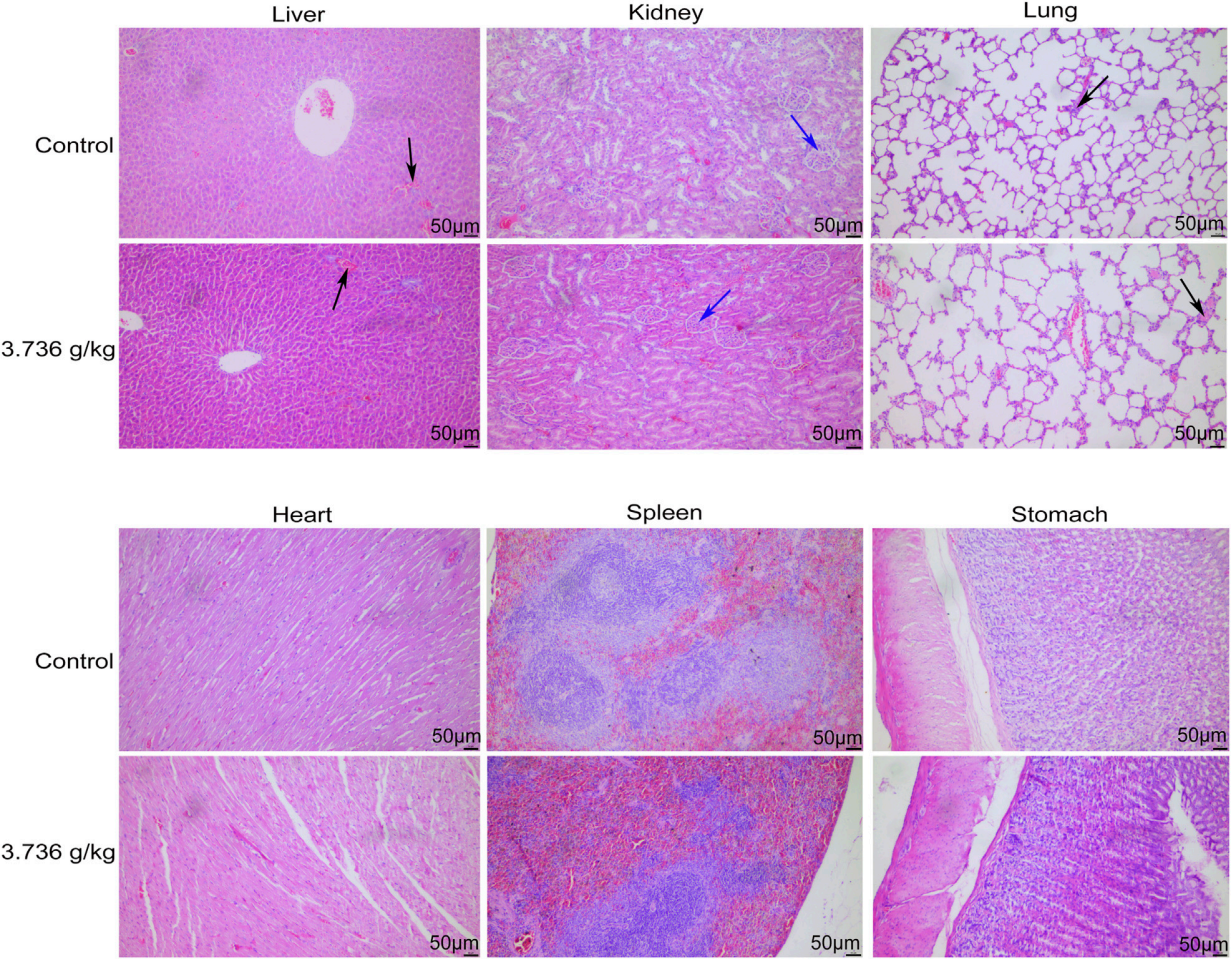
Effects of 180 days of oral treatment with ZRH on histopathological parameters of female rats. H&E staining (×20) of the liver of control and 3.736 g/kg; kidney of control and 3.736 g/kg; lung of control and 3.736 g/kg; heart of control and 3.736 g/kg; spleen of control and 3.736 g/kg; stomach of control and 3.736 g/kg of the female rats, scale bar = 50 μm. The black arrow indicates spontaneous inflammatory cell infiltration including lung/liver, while the blue arrow indicates calcium deposition in renal tubules.

When gross necropsies were conducted, we detected no evidence of pathological lesions in untreated or treated rats. As such, histopathological analyses were only conducted using tissue samples from untreated controls and rats treated with 3.736 g/kg of ZRH. The only lesions detected were common lesions that spontaneously develop in SD rats including inflammatory cell infiltration of the lung/liver and calcium salt deposition in the renal epithelium, and these lesions were present in rats from both analyzed groups. No histopathological changes were observed in the liver, kidney, lung, heart, stomach, spleen and other organs in the control or 3.736 g/kg treatment groups for both male (Figure 5) and female (Figure 6) rats.

### 3.4 Effects of ZRH on transcript profiling of liver in rats in a 180-day repeated oral toxicity study

**FIGURE 7:**
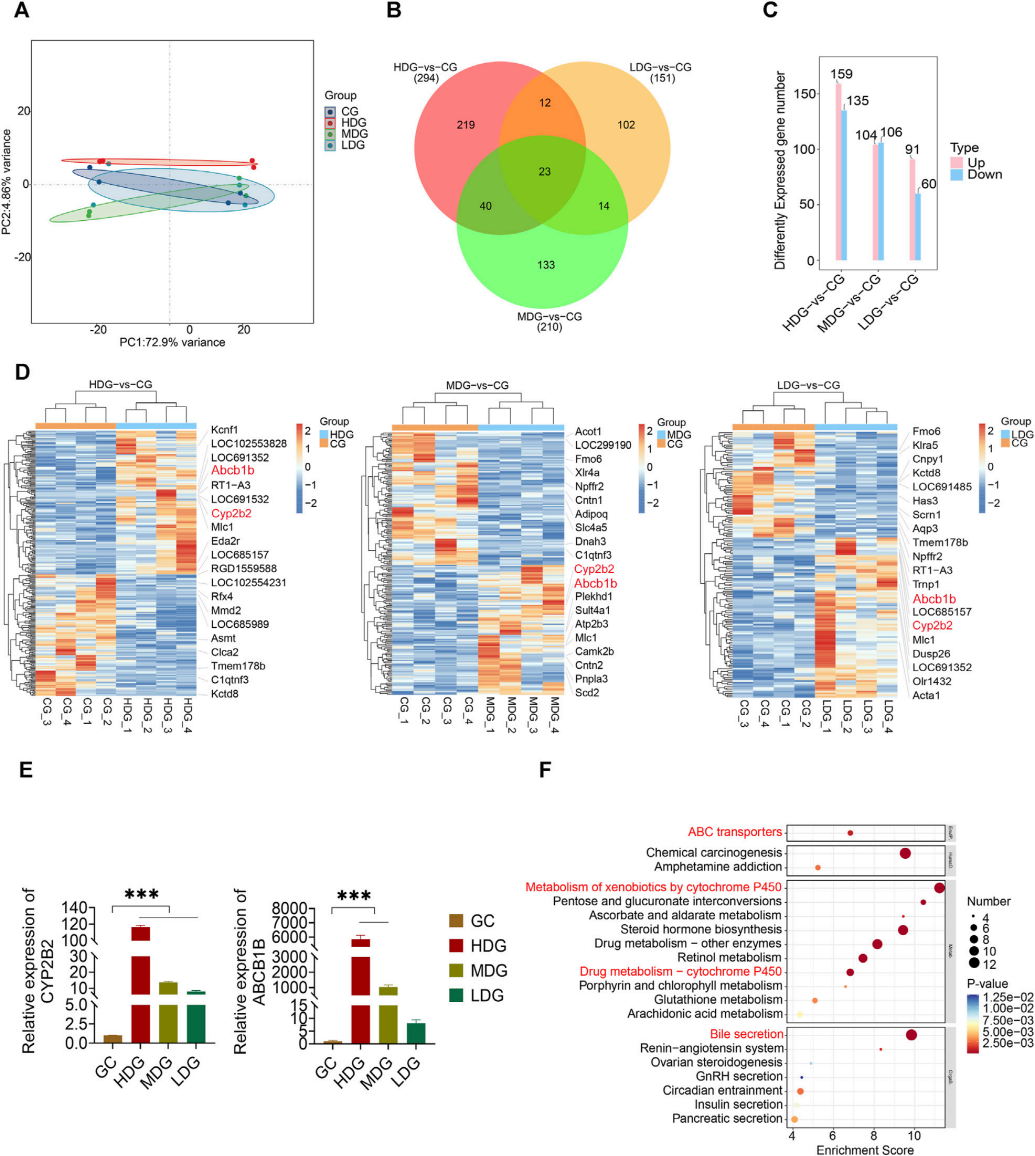
Effects of ZRH on transcript profiling of liver in rats in a 180-day repeated oral toxicity study. **(A)** PCA analysis of the differentially expressed genes in each group of SD rats. **(B)** Venn diagram of significantly enriched DEGs in different group comparisons represented the unique and overlapping DEGs. **(C)** Employing a significance threshold of *p* < 0.05 and an absolute LogFC (fold change) > 1, differentially expressed genes (DEGs) were identified. **(D)** Heat map analysis of DEGs between two groups including HDG and CG, MDG and CG, LDG and CG. **(E)** The mRNA levels of 2 genes including *Abcb1b* and *Cyp2b2*, were determined by real-time PCR. **(F)** KEGG database analysis was conducted to assess the biological functions of DEGs (CG-vs HDG, MDG, and LDG) and the top 20 significant pathways were presented using bubble plots. ^***^ *p* < 0.001.

Transcriptomics analysis was conducted on liver tissues to investigate alterations resulting from ZRH administration in a 180-day repeated oral toxicity study. Principal Component Analysis (PCA) revealed that the first and second components accounted for 72.9% and 4.86% of the variance, respectively (Figure 7A). Employing a significance threshold of *p* < 0.05 and an absolute LogFC (fold change) > 1, differentially expressed genes (DEGs) were identified. Comparing the high dose group (HDG) to control group, 294 gene sets were significantly altered (Figure 7B; Supplemetary Table S7), with 159 upregulated and 135 downregulated genes (Figure 7C; Supplementary Table S7). Similarly, in comparison with the medium dose group (MDG), 210 gene sets were remarkably altered (Figure 7B; Supplementary Table S7), comprising 104 upregulated and 106 down-regulated genes (Figure 7C; Supplementary Table S7). In the low dose group (LDG), 151 genes showed differential expression (Figure 7B; Supplementary Table S7), with 91 up-regulated and 60 down-regulated genes (Figure 7C; Supplementary Table S7). To visualize the significant DEGs, a heatmap generated by hierarchical clustering was employed (Figure 7D), highlighting substantial differences in gene expression across the groups. Among the HDG, MDG, and LDG groups, 23 DEGs were common, including *Abcb1b*, *Bhlha15*, *C1qtnf3*, *Camk2b*, *Ccdc57*, *Cenpp*, *Cyp2b2*, *Dusp26*, *Evc2*, *Gadl1*, *Gdnf*, *Kcnk2*, *Kctd8*, *LOC108350501*, *Lilrb3*, *Ly6al*, Mlc1, *Npffr2*, *Rps12l2*, *Timd4*, *Tmem178b*, *Tmtc2* and *Tpsb2* (Figure 7D). These shared DEGs, such as *Abcb1b* and *Cyp2b2*, known for its role in liver drug transport and metabolism function, might be pivotal in the context of the 180-day repeated oral toxicity study treated with ZRH. Furthermore, the transcriptomic analysis indicated prominent upregulation of critical genes like *Abcb1b* and *Cyp2b2* upon ZRH treatment. This observation was also confirmed by the results qPCR of liver tissue in various ZRH treatment groups (HDG, MDG, and LDG), where *Abcb1b* and *Cyp2b2* expression was substantially increased. Conversely, *Abcb1b* and *Cyp2b2* expression was notably reduced in the control group (CG) (Figure 7E). To elucidate potential pathways influenced by ZRH, a KEGG database analysis was conducted to assess the biological functions of DEGs (CG-vs HDG, MDG, and LDG). The top 20 significant pathways were presented using bubble plots (Figure 7F). In comparison with the HDG, MDG, and LDG groups, the pathways influenced by ZRH were primarily associated with liver drug metabolism, including ABC transporters, metabolism of xenobiotics by cytochrome P450, drug metabolism via other enzymes, drug metabolism via cytochrome P450, glutathione metabolism, and bile secretion (Figure 7F).

### 3.5 Effects of ZRH on metabolite profiling of liver in rats in a 180-day repeated oral toxicity study

**FIGURE 8:**
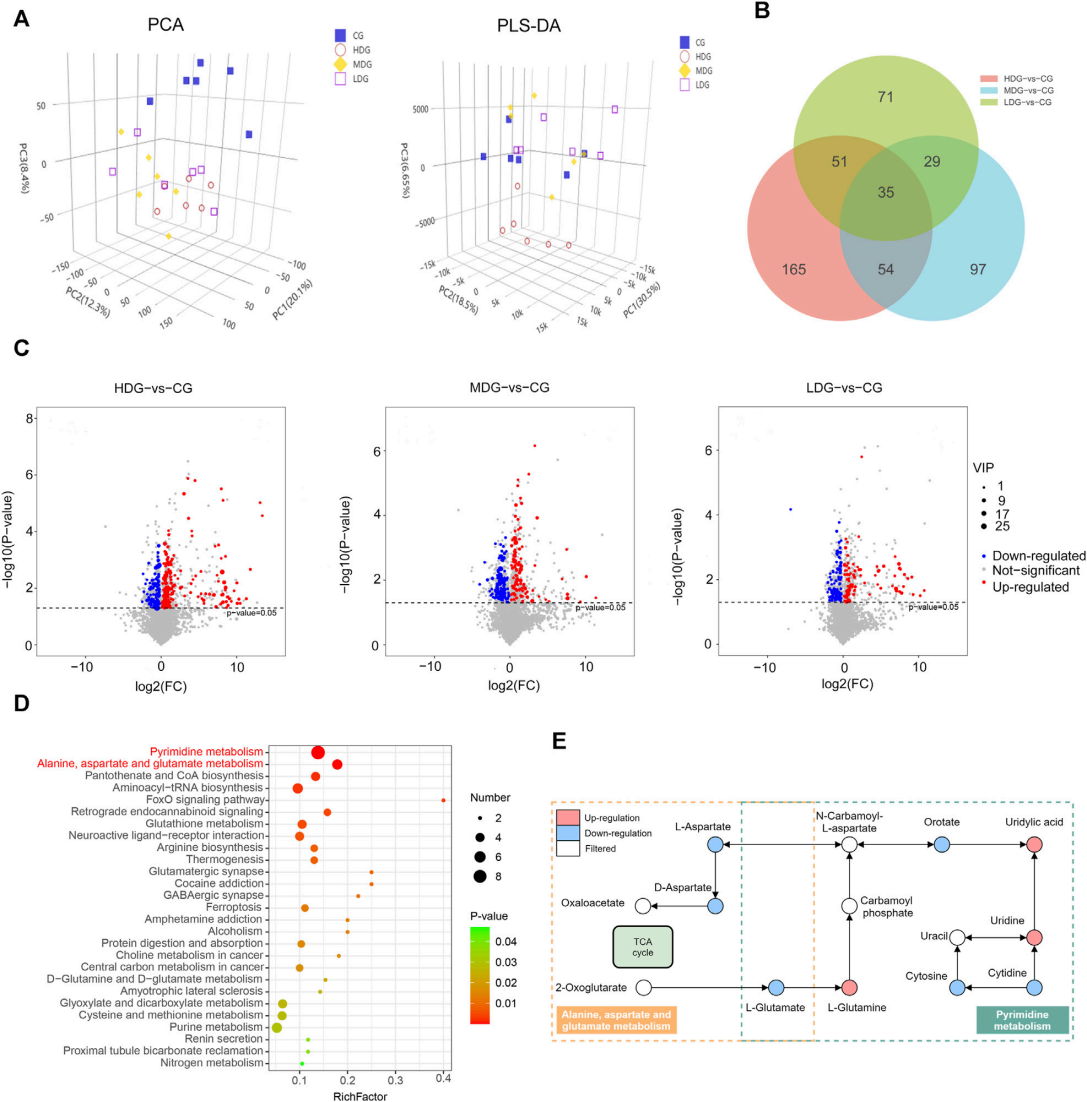
Effects of ZRH on metabolite profiling of liver in rats in a 180-day repeated oral toxicity study. **(A)** PCA and PLS-DA score plots of different groups including CG, MDG, MDG and HDG. **(B)** Venn diagram of significantly enriched DAMs in different group comparisons represented the unique and overlapping DAMs. **(C)** These DAMs were visualized using volcano plots . **(C)** KEGG database analysis was conducted to assess the biological functions of DAMs (CG-vs HDG, MDG, and LDG) and the top significant pathways were presented using bubble plots. **(D)** This network visualization provides a comprehensive understanding of how ZRH treatment influences cellular metabolism and the potential implications for various metabolic pathways. **(E)** Based on the DAMs potentially linked to ZRH treatment (CG-vs HDG, MDG, and LDG), a metabolic network was constructed using the KEGG database to illustrate the altered metabolic pathways induced by ZRH. The differential metabolites were measured by the combination of the PLS-DA model (VIP > 1) and the two-tailed Student’s t-test (P < 0.05) on the normalized peak intensities.

To investigate the impact of ZRH treatment on cellular responses, we conducted metabolomic analyses on samples exposed to ZRH and control liver samples. This was done using LC-MS/MS in both positive and negative ion modes. The quality control (QC) samples demonstrated good reproducibility and reliability, as depicted in the PCA score plot (Figure 8A). Further more, the PCA plot indicated clear separation between the control group (CG) and the other groups (HDG, MDG, and LDG). To account for potential confounding variables unrelated to group differences, PLS-DA analysis was employed (Figure 8B). This analysis successfully distinguished the four distinct groups, indicating a robust discrimination model. Using criteria of VIP >1 and *p*-value <0.05 in the PLS-DA model, differentially accumulated metabolites (DAMs) were identified. These DAMs were visualized using volcano plots (Figure 8C). When comparing the high-dose group (HDG) to the control group, 305 DAMs showed significant alterations, with 205 upregulated and 100 downregulated metabolites. Similarly, the medium-dose group (MDG) exhibited 215 DAMs, including 104 upregulated and 111 downregulated metabolites. In the low-dose group (LDG), 186 DAMs were identified, with 93 upregulated and 93 downregulated metabolites (Supplementary Table S8). To gain insight into the functions of these differentially regulated metabolites, all DAMs from the comparisons (CG-vs HDG, MDG, and LDG) were mapped to the KEGG database. The top enriched pathways, as shown in Figure 8D, were primarily related to pyrimidine metabolism, as well as alanine, aspartate, and glutamate metabolism. Based on the DAMs potentially linked to ZRH treatment (CG-vs HDG, MDG, and LDG), a metabolic network was constructed using the KEGG database to illustrate the altered metabolic pathways induced by ZRH (Figure 8E). This network visualization provides a comprehensive understanding of how ZRH treatment influences cellular metabolism and the potential implications for various metabolic pathways. In the context of pyrimidine metabolism, exposure to ZRH led to notable down-regulation of metabolites such as L-glutamate, orotate, cytosine, and cytidine. Conversely, there was a marked up-regulation of metabolites like L-glutamine, uridylic acid, and uridine as a result of ZRH treatment. Shifting focus to the alanine, aspartate, and glutamate metabolism pathway, ZRH treatment primarily induced a significant down-regulation of L-glutamate, D-aspartate, and L-aspartate.

## 4 Discussion

ZRH has long been used as a traditional Mongolian medicinal preparation wherein it is employed to treat CHD (Mu et al., 2019), and to improve immune function owing to its ability to calm the mind and to benefit qi and the spleen and to calm the mind. Rates of traditional medicine use are rising throughout the world, raising concerns regarding the often poorly understood nephrotoxicity and hepatotoxicity profiles of these medicines, many of which have not been evaluated in clinical trials (Bahmani et al., 2014; Lin et al., 2015; Cui et al., 2019). The first priority in herbal medicine is assessment of the toxic characteristics of herbal products. For selecting a safe dose in human clinical setting, systematic toxicological studies must be performed using different experimental models (e.g., ames test, comet assay, MTT assay and animal studies). Toxicity effects of herbal products humans or animals ascertain in the form of adverse histopathological, haematological, serum biochemistry, cardiovascular or gastrointestinal effects, structural rearrangements caused by DNA damage. As such, a controlled assessment of ZRH was necessary to identify a safe dose of this traditional medicine. Toxicity studies can be acute, subchronic or chronic in design (Li et al., 2020). Herein, we performed acute and 180-day repeated oral toxicity studies of ZRH to better understand its possible toxicity profile.

Despite its positive regulation in CHD treatment, the chemical compounds of ZRH remains elusive, hindering any further explanation of its toxicological mechanism. Thus, we first analyzed the chemical compounds of ZRH by the HPLC-Q-Exactive-MS/MS analysis. Furthermore, lignans, flavonoids, phenylpropanoids, organic acids, and phenolic acids, constituted the main clusters in the high-resolution mass spectrometry analysis, providing evidence that these compounds are major components of ZRH.

Next, we assessed the acute and the 180-day repeated oral toxicity study of ZRH using KM mice and SD rats, respectively. LD50 (an estimation of toxicant or agents to subjects) is the first step to be conducted assessment of the toxic effects of herbal medicine. Many indices of potential types of drug activity (Williams et al., 2000) were provided according to the LD50 values. In the acute toxicity assay, oral treatment with ZRH was well tolerated. In fact, the limit test (maximum administration volume and maximum drug concentration) is not only intended for determining LD50 value of plant extracts, but also it serves as a suggestion for classifying the plant extract (Shafaei et al., 2015). According to the Globally Harmonized System of Classification of Chemical Substances and Mixtures (GSH) adopted by the OECD, this plant extract can therefore be considered a Class 5 drug and non-toxic substance (OECD, 2001). Since no toxicological reaction was found during the acute toxicity study period in mice, further research was conducted to assess the 180-day toxicity in rats to prepare the comprehensive toxicology data of ZRH.

Analyzing serum biochemistry and hematological parameters can offer insight into the effects of a given drug on organ functionality. Hematological parameters provide important information regarding the blood of a treated organism, while measures of liver and kidney function are crucial given that both organs are essential for survival (Malin et al., 2019). Liver damage can be measured in a sensitive manner by evaluating circulating ALT, AST, ALP, and GGT enzyme levels (Huang et al., 2019), while TP can be assessed to estimate nutritional status and to diagnose liver and kidney diseases (Javadi et al., 2019), and serum urea and CRE levels are reflective of potential renal dysfunction (McCabe et al., 2016; Lotti and Maggi, 2018). Almost all biochemical parameters analyzed remained within the reference levels for the species. We detected a dose-dependent increase in UREA levels in female rats at the end of the treatment period, although it is possible that this effect is associated with dehydration owing to insufficient water intake. We also observed transient changes in hematological (EOS %, HTC, HGB, and MCHC) and biochemical (K^+^, TG, GLU, UREA, GGT, and TP) parameters in treated rats, but these variations were within the normal range and were not observed in the rats treated with higher or lower doses, indicating that they were not related with ZRH treatment over the course of this 180-day repeated oral toxicity study. The lack of alteration in the liver parameters (ALT, AST, ALP) and indicators of kidney function (CREA, TP, ALB) showed that the administration of ZRH for 180 days at dose of 0.934, 1.868, or 3.736 g/(kg·d) did not cause any abnormal changes as reflected by the liver and renal function tests.

Histopathological analyses of major organs from animals in the control and 3.736 g/kg treatment groups also confirmed above findings. In general, microscopic damage to the liver, heart, or kidneys can be detected via light microscopy and will tend to coincide with abnormal serum biochemistry findings, suggesting the potential for drug-related toxicities. However, we observed no abnormal histological findings when comparing samples from these treatment and control groups even at the highest tested ZRH dose. Since there were no obvious histological abnormality observed in liver and kidney, therefore strongly suggested that there were no obvious detrimental effects or morphological disturbances caused by the daily oral administration of ZRH for 180 days, even at the highest tested dose of 3.736 g/kg.

Since the 180-day repeated oral toxicity study provides some indication of repeated exposure to plant extract or chemical compound over a fraction of the average lifespan of laboratory animals (Zuo et al., 2021). Specifically, they provide information on target organ toxicity and also help determine appropriate dosing regimens for long-term use. In the 180-day repeated oral toxicity study, we observed no significant ZRH-related mortality or adverse events at doses of 0.934 g/kg, 1.868 g/kg, and 3.736 g/kg, which are roughly 20-, 40-, and 80-fold higher than the doses used by humans in clinical settings respectively. Relative organ weights were observed in these toxicity studies to be a relatively sensitive indicator of specific organs, therefore, toxicity was defined as significant changes observed in these specific organs (Shafaei et al., 2015). In our study, we did observe significant changes in the relative liver weight ratio in both male and female rats at the end of the study period relative to control rats. These results indicate that long-term use of ZRH may have potential liver toxicity. In order to comprehensively elucidate the underlying mechanisms behind the observed toxicity of ZRH on rat livers, we conducted a comprehensive analysis by integrating transcriptomic and metabolomic data.

In the context of a 180-day repeated oral toxicity study, our transcriptomic analysis conducted on rat livers provided direct evidence of the alterations in gene expression levels induced by ZRH. The results of our study highlighted the extensive impact of ZRH on gene expression within the rat liver. Specifically, our findings revealed that ZRH had a substantial effect on key pathways including ABC transporters, metabolism of xenobiotics by cytochrome P450, drug metabolism via other enzymes, drug metabolism via cytochrome P450, glutathione metabolism, and bile secretion. Within these pathways, critical genes played a crucial role in liver drug transport and metabolism functions, such as *Abcb1b* and *Cyp2b2*. The genes *Abcb1a* and *Abcb1b* encode the rat proteins *ABCB1A* and *ABCB1B*, respectively, which represent two isoforms of rat P-glycoprotein (Su et al., 2017). These isoforms collectively exhibit functional characteristics similar to those of the human *ABCB1* protein, commonly referred to as human P-glycoprotein. These rat proteins, like their human counterpart, are part of the ABC transporter superfamily and play a crucial role in actively transporting a diverse array of substances and drugs out of cells (Su et al., 2017). The genes *Cyp2b1* and *Cyp2b2* encode cytochrome P450 enzymes, specifically belonging to the *CYP2B* family. These enzymes play a pivotal role in metabolism. Notably, the *Cyp2b* enzymes are expressed in both lung and liver tissues across a range of animal species, including rat (*Cyp2b10*), rat (*Cyp2b1* and *Cyp2b2*), rabbit (*Cyp2b4*), dog (*Cyp2b11*), and human (*Cyp2b6*) (Villard et al., 1998). In rats, these two isoforms exhibit distinct tissue-specific expression patterns:*Cyp2b2* is primarily prevalent in lung tissue, with comparatively lower levels in the liver, whereas *Cyp2b2* predominates in the liver (Desrochers et al., 1996). This differentiation in expression locations highlights the specialized functions these enzymes perform in various contexts.The *CYP2B* family is renowned for its capacity to metabolize a diverse spectrum of substances, encompassing endogenous compounds, pharmaceutical agents, and environmental pollutants (Wojtczak and Skretkowicz, 2009). Given this extensive role, it is imperative to consider the effects of these isoforms’ genetic variations when designing pharmaceuticals and assessing individual susceptibilities to environmental toxins. As a result, understanding these polymorphisms holds significance not only in pharmaceutical development but also in comprehending personal responses to environmental hazards. The insights garnered from investigating these genes and their isoforms can be instrumental in advancing drug design and optimizing therapeutic strategies, as well as in safeguarding individuals from adverse effects linked to drug and environmental contaminants.

Metabolomic analyses offer a powerful approach to identify metabolites that have the potential to modulate a wide array of biological processes, thereby influencing the phenotypes of cells or organisms (Guijas et al., 2018). In our study, we conducted comprehensive metabolomic analyses on liver samples exposed to ZRH, revealing significant alterations in metabolite levels. Enrichment analysis unveiled pronounced disruptions in essential metabolic pathways, including pyrimidine metabolism, as well as alanine, aspartate, and glutamate metabolism, all of which play pivotal roles in liver drug metabolism. One intriguing aspect is the potential interplay between pyrimidine metabolism and drug metabolism. Specifically, certain drug-metabolizing enzymes, notably the cytochrome P450 (CYP450) enzymes, depend on cofactors like NADPH for their enzymatic activity (Zhu and Lee, 2005). Elevated pyrimidine synthesis, such as during tissue regeneration, could lead to an increased demand for NADPH (Smith et al., 2023). This heightened demand might indirectly impact the availability of NADPH for drug metabolism, consequently influencing drug clearance dynamics (Smith et al., 2023). Moreover, both pyrimidine metabolism and drug metabolism share common substrates, including amino acids and intermediates from diverse metabolic pathways. The heightened demand for these substrates during pyrimidine synthesis might potentially limit their availability for drug metabolism, potentially affecting the overall efficiency of drug clearance. In the context of pyrimidine metabolism, exposure to ZRH resulted in noticeable downregulation of metabolites such as L-glutamate, orotate, cytosine, and cytidine. Intriguingly, a distinct upregulation was observed in metabolites like L-glutamine, uridylic acid, and uridine following ZRH treatment. These findings strongly suggest that these specific metabolites could serve as potential biomarkers closely associated with ZRH-induced hepatotoxicity, as observed in our 180-day repeated oral toxicity study. In summary, our metabolomic analyses shed light on the intricate connections between pyrimidine metabolism as well as alanine, aspartate, and glutamate metabolism and liver drug metabolism, underscoring their potential influence on drug clearance and toxicity. The alterations in metabolite levels observed following ZRH exposure not only provide valuable insights into the mechanisms underlying hepatotoxicity but also unveil potential biomarkers for assessing drug-induced liver damage.

This study offers comprehensive insights into metabolic and transcriptional changes as well as key genes and metabolites underlying the response to ZRH exposure, which provided key clues for further study on the toxicological mechanisms of ZRH. Although great effort has been made, quantitative ZRH’s safety evaluation is still progressing. Future areas may include the use of developmental toxicity, carcinogenicity, cytotoxicity and safey pharmacology in different models (e.g., Ames test, Comet assay, MTT assay, etc.). Moving forward, as a new drug development, we will continue to promote the use of best methods to address ZRH’s safety, which are appropriate, sound, and efficient.

## 5 Conclusion

In the realm of these discoveries, our study unveils a panoramic understanding of the temporal, dosage-specific, and gene dimensions surrounding the metabolic and transcriptional shifts induced by ZRH exposure. Delving into pivotal genes and metabolites, our findings establish a resilient groundwork, paving the way for the secure integration of ZRH in clinical treatments for patients. As we peer into the future, recommendations emerge for further exploration, encompassing aspects such as time dynamics, dosage considerations, and gene-centric avenues to enhance therapeutic efficacy.

## Data Availability

The datasets presented in this study can be found in online repositories. The names of the repository/repositories and accession number(s) can be found below: NCBI Sequence Read Archive (SRA) database (PRJNA1041985).
