# Supplementary material for: Integration of transcriptomics and metabolomics reveals toxicological mechanisms of ZhuRiHeng drop pill in the 180-day repeated oral toxicity study
Source: Front Pharmacol. 2024 Mar 15;15:1333167. doi: 10.3389/fphar.2024.1333167 (PMC10978746; doi:10.3389/fphar.2024.1333167)
Supplement: Supplementary file 1 [file Table4.DOC]

**Table S4.** Hematological analysis of female SD rats during 180-day repeated oral toxicity study.

| Time  point | Parameters | Groups | | | |
| --- | --- | --- | --- | --- | --- |
|  |  | Control | 0.934 g/kg | 1.868 g/kg | 3.736 g/kg |
| D91  (mid-dosing period) | RBC (×10^12^/L) | 7.50 ± 0.22 | 7.54 ± 0.17 | 7.56 ± 0.28 | 7.49 ± 0.52 |
|  | HCT (%) | 39.5 ± 0.7 | 39.4 ± 1.6 | 39.9 ± 1.0 | 39.7 ± 1.9 |
|  | MCV (fL) | 52.7 ± 1.2 | 52.3 ± 1.4 | 52.9 ± 1.5 | 53.1 ± 1.7 |
|  | HGB (g/L) | 137 ± 3 | 135 ± 4 | 138 ± 4 | 137 ± 8 |
|  | MCH (pg) | 18.3 ± 0.3 | 17.9 ± 0.4 | 18.2 ± 0.5 | 18.3 ± 0.3 |
|  | MCHC (g/L) | 346 ± 3 | 343 ± 5 | 345 ± 2 | 344 ± 7 |
|  | PLT (×10^9^/L) | 780 ± 99 | 819 ± 66 | 813 ± 100 | 791 ± 84 |
|  | WBC (×10^9^/L) | 1.666 ± 0.524 | 1.864 ± 0.839 | 2.058 ± 0.776 | 1.792 ± 0.454 |
|  | LYM % | 77.6 ± 5.6 | 71.1 ± 8.7 | 74.3 ± 6.4 | 73.8 ± 8.0 |
|  | NEUT % | 18.6 ± 5.4 | 25.4 ± 7.9 | 21.6 ± 5.7 | 22.5 ± 8.6 |
|  | EOS % | 2.288 ± 0.885 | 1.888 ± 0.585 | 2.200 ± 0.667 | 1.808 ± 1.183 |
|  | BASO % | 0.000 ± 0.000 | 0.000 ± 0.000 | 0.000 ± 0.000 | 0.000 ± 0.000 |
|  | MONO % | 1.588 ± 0.574 | 1.628 ± 0.415 | 1.840 ± 0.948 | 1.948 ± 0.700 |
|  | RET % | 2.49 ± 0.58 | 2.96 ± 0.77 | 3.32 ± 1.23 | 2.97 ± 0.85 |
|  | PT (s) | 18.5 ± 1.7 | 18.7 ± 1.2 | 17.1 ± 1.3 | 17.9 ± 1.1 |
|  | APTT (s) | 19.9 ± 0.4 | 20.2 ± 2.0 | 19.6 ± 0.6 | 19.1 ± 0.5 |
|  | TT (s) | 25.5 ± 1.8 | 26.6 ± 1.2 | 25.1 ± 2.5 | 26.3 ± 2.1 |
|  | FIB (g/L) | 1.44 ± 0.31 | 1.30 ± 0.13 | 1.30 ± 0.04 | 1.43 ± 0.26 |
| D182  (end-dosing period) | RBC (×10^12^/L) | 7.44 ± 0.32 | 7.28 ± 0.41 | 7.13 ± 0.28 | 7.13 ± 0.33 |
|  | HCT (%) | 39.4 ± 0.7 | 38.2 ± 1.4 | 38.7 ± 1.5 | 38.6 ± 1.9 |
|  | MCV (fL) | 53.0 ± 2.1 | 52.5 ± 1.9 | 54.3 ± 2.0 | 54.2 ± 2.1 |
|  | HGB (g/L) | 136 ± 3 | 133 ± 6 | 133 ± 6 | 133 ± 7 |
|  | MCH (pg) | 18.4 ± 0.6 | 18.3 ± 0.5 | 18.7 ± 0.6 | 18.6 ± 0.7 |
|  | MCHC (g/L) | 346 ± 4 | 348 ± 5 | 345 ± 7 | 343 ± 2 |
|  | PLT (×10^9^/L) | 751 ± 106 | 824 ± 114 | 828 ± 112 | 821 ± 93 |
|  | WBC (×10^9^/L) | 2.69 ± 0.86 | 2.83 ± 0.77 | 2.83 ± 0.93 | 2.73 ± 0.54 |
|  | LYM % | 67.9 ± 10.0 | 75.6 ± 10.0 | 76.5 ± 7.4 | 75.1 ± 5.9 |
|  | NEUT % | 26.71 ± 10.92 | 19.84 ± 9.63 | 18.07 ± 6.20 | 18.58 ± 4.83 |
|  | EOS % | 1.880 ± 0.694 | 1.780 ± 0.413 | 1.960 ± 1.047 | 2.210 ± 0.860 |
|  | BASO % | 0.000 ± 0.000 | 0.000 ± 0.000 | 0.000 ± 0.000 | 0.000 ± 0.000 |
|  | MONO % | 3.55 ± 1.06 | 2.76 ± 0.81 | 3.45 ± 1.35 | 4.14 ± 1.97 |
|  | RET % | 3.15 ± 0.51 | 3.07 ± 0.67 | 3.66 ± 0.64 | 3.34 ± 0.67 |
|  | PT (s) | 18.2 ± 2.8 | 18.5 ± 2.2 | 18.7 ± 2.1 | 20.0 ± 2.0 |
|  | APTT (s) | 20.7 ± 1.8 | 20.9 ± 3.0 | 19.4 ± 1.0 | 19.7 ± 1.1 |
|  | TT (s) | 31.4 ± 3.3 | 30.8 ± 2.8 | 30.0 ± 2.2 | 31.6 ± 3.0 |
|  | FIB (g/L) | 1.225 ± 0.213 | 1.165 ± 0.264 | 1.328 ± 0.270 | 1.313 ± 0.335 |
| D210  (recovery period) | RBC (×10^12^/L) | 7.68 ± 0.41 | 7.84 ± 0.52 | 7.55 ± 0.35 | 7.78 ± 0.73 |
|  | HCT (%) | 40.6 ± 1.4 | 40.9 ± 2.7 | 39.3 ± 1.5 | 40.6 ± 3.6 |
|  | MCV (fL) | 52.9 ± 1.9 | 52.2 ± 1.1 | 52.1 ± 1.8 | 52.2 ± 1.3 |
|  | HGB (g/L) | 138 ± 6 | 140 ± 7 | 135 ± 5 | 139 ± 14 |
|  | MCH (pg) | 18.0 ± 0.4 | 17.9 ± 0.3 | 17.9 ± 0.6 | 17.9 ± 0.4 |
|  | MCHC (g/L) | 341 ± 6 | 342 ± 6 | 344 ± 4 | 344 ± 5 |
|  | PLT (×10^9^/L) | 757 ± 62 | 732 ± 39 | 780 ± 55 | 819 ± 100 |
|  | WBC (×10^9^/L) | 2.83 ± 0.61 | 1.99 ± 0.74 | 2.30 ± 0.53 | 2.35 ± 1.37 |
|  | LYM % | 77.2 ± 5.2 | 75.7 ± 2.9 | 72.1 ± 10.4 | 70.4 ± 7.1 |
|  | NEUT % | 18.18 ± 4.37 | 18.86 ± 3.44 | 22.94 ± 10.33 | 22.38 ± 5.92 |
|  | EOS % | 1.82 ± 0.65 | 2.16 ± 0.34 | 1.96 ± 0.36 | 2.36 ± 0.95 |
|  | BASO % | 0.000 ± 0.000 | 0.000 ± 0.000 | 0.000 ± 0.000 | 0.000 ± 0.000 |
|  | MONO % | 2.84 ± 1.23 | 3.24 ± 1.02 | 3.02 ± 0.69 | 4.84 ± 2.35 |
|  | RET % | 2.49 ± 0.50 | 2.69 ± 0.37 | 2.71 ± 0.36 | 2.55 ± 0.28 |
|  | PT (s) | 21.5 ± 2.2 | 22.1 ± 2.4 | 22.6 ± 1.6 | 21.3 ± 1.3 |
|  | APTT (s) | 17.7 ± 1.1 | 18.8 ± 1.0 | 18.5 ± 0.5 | 19.0 ± 1.3 |
|  | TT (s) | 24.8 ± 1.0 | 24.5 ± 0.8 | 24.7 ± 1.4 | 25.6 ± 2.5 |
|  | FIB (g/L) | 1.232 ± 0.123 | 1.272 ± 0.110 | 1.242 ± 0.166 | 1.142 ± 0.178 |

Data are expressed as mean ± SD with one-way ANOVA followed by the LSD multiple comparisons test, statistically significant compared to control (^*^*P* < 0.05, ^**^*P* < 0.01, ^***^*P* < 0.001; D91 *n* = 5, D182 *n* = 10, D210 *n* = 5)
