# Supplementary material for: Integration of transcriptomics and metabolomics reveals toxicological mechanisms of ZhuRiHeng drop pill in the 180-day repeated oral toxicity study
Source: Front Pharmacol. 2024 Mar 15;15:1333167. doi: 10.3389/fphar.2024.1333167 (PMC10978746; doi:10.3389/fphar.2024.1333167)
Supplement: Supplementary file 2 [file Table6.DOC]

**Table S6.** Serum biochemistry analysis of female SD rats during 180-day repeated oral toxicity study.

| Time  point | Parameters | Groups | | | |
| --- | --- | --- | --- | --- | --- |
|  |  | Control | 0.934 g/kg | 1.868 g/kg | 3.736 g/kg |
| D91  (mid-dosing period) | ALT (U/L) | 51.2 ± 29.3 | 36.0 ± 9.4 | 36.4 ± 2.7 | 34.2 ± 7.4 |
|  | AST (U/L) | 130.0 ± 49.9 | 94.8 ± 21.8 | 89.0 ± 14.2 | 80.0 ± 21.9 |
|  | ALP (U/L) | 64.8 ± 25.5 | 63.8 ± 14.0 | 72.2 ± 18.1 | 65.4 ± 27.6 |
|  | CK (U/L) | 377 ± 161 | 320 ± 128 | 352 ± 97 | 343 ± 159 |
|  | TBIL (μmol/L) | 1.140 ± 0.251 | 0.920 ± 0.084 | 1.080 ± 0.192 | 1.080 ± 0.311 |
|  | TP (g/L) | 59.0 ± 5.7 | 57.3 ± 3.4 | 59.4 ± 1.7 | 59.0 ± 4.6 |
|  | ALB (g/L) | 35.0 ± 3.5 | 34.0 ± 1.8 | 35.2 ± 0.7 | 34.8 ± 2.1 |
|  | GLU (mmol/L) | 6.23 ± 0.35 | 6.69 ± 0.71 | 6.66 ± 0.22 | 6.87 ± 0.38 |
|  | UREA (mmol/L) | 7.02 ± 0.74 | 7.22 ± 1.30 | 6.46 ± 0.61 | 7.16 ± 1.54 |
|  | CRE (μmol/L) | 54.4 ± 3.9 | 52.7 ± 4.3 | 53.0 ± 4.2 | 49.8 ± 2.5 |
|  | TCHO (mmol/L) | 2.20 ± 0.35 | 2.38 ± 0.63 | 2.43 ± 0.19 | 2.88 ± 0.62 |
|  | TG (mmol/L) | 0.428 ± 0.113 | 0.344 ± 0.056 | 0.348 ± 0.152 | 0.432 ± 0.091 |
|  | GGT (U/L) | 0.494 ± 0.138 | 0.410 ± 0.293 | 0.616 ± 0.172 | 0.786 ± 0.358 |
|  | K^+^ (mmol/L) | 3.46 ± 0.18 | 3.51 ± 0.18 | 3.74 ± 0.15^*^ | 3.94 ± 0.22^**^ |
|  | Na^+^ (mmol/L) | 145 ± 2 | 145 ± 2 | 144 ± 2 | 144 ± 1 |
|  | Cl^+^ (mmol/L) | 106 ± 1 | 107 ± 2 | 106 ± 0 | 107 ± 2 |
| D182  (end-dosing period) | ALT (U/L) | 52.7 ± 41.9 | 42.1 ± 8.1 | 54.5 ± 30.7 | 37.8 ± 11.6 |
|  | AST (U/L) | 110.3 ± 33.0 | 85.1 ± 17.1 | 98.3 ± 34.1 | 92.4 ± 20.5 |
|  | ALP (U/L) | 48.5 ± 17.2 | 68.3 ± 19.4 | 54.2 ± 21.0 | 51.7 ± 18.2 |
|  | CK (U/L) | 263.5 ± 83.9 | 203.3 ± 69.3 | 259.8 ± 137.2 | 251.9 ± 104.6 |
|  | TBIL (μmol/L) | 0.800 ± 0.521 | 0.578 ± 0.504 | 0.650 ± 0.422 | 0.820 ± 0.286 |
|  | TP (g/L) | 71.7 ± 4.4 | 68.6 ± 3.1 | 68.5 ± 4.9 | 66.3 ± 3.7^**^ |
|  | ALB (g/L) | 36.9 ± 2.8 | 35.2 ± 1.9 | 35.1 ± 2.5 | 34.2 ± 2.0 |
|  | GLU (mmol/L) | 6.36 ± 0.56 | 6.63 ± 0.78 | 6.09 ± 0.48 | 6.24 ± 0.82 |
|  | UREA (mmol/L) | 5.40 ± 0.75 | 7.17 ± 1.21^**^ | 7.26 ± 1.10^***^ | 7.10 ± 0.94^**^ |
|  | CRE (μmol/L) | 45.8 ± 3.7 | 44.8 ± 5.5 | 43.4 ± 3.7 | 45.1 ± 7.6 |
|  | TCHO (mmol/L) | 2.16 ± 0.45 | 2.14 ± 0.21 | 2.24 ± 0.45 | 2.23 ± 0.43 |
|  | TG (mmol/L) | 0.950 ± 0.478 | 0.644 ± 0.256 | 0.761 ± 0.348 | 0.689 ± 0.465 |
|  | GGT (U/L) | 0.260 ± 0.130 | 0.363 ± 0.200 | 0.443 ± 0.206^*^ | 0.509 ± 0.187^**^ |
|  | K^+^ (mmol/L) | 3.59 ± 0.18 | 3.71 ± 0.12 | 3.77 ± 0.29 | 4.02 ± 0.19^***^ |
|  | Na^+^ (mmol/L) | 142 ± 1 | 142 ± 1 | 143 ± 1 | 142 ± 2 |
|  | Cl^+^ (mmol/L) | 105 ± 2 | 105 ± 1 | 107 ± 1 | 105 ± 2 |
| D210  (recovery period) | ALT (U/L) | 57.6 ± 21.9 | 39.0 ± 10.4 | 52.0 ± 20.3 | 43.4 ± 18.6 |
|  | AST (U/L) | 110.2 ± 10.1 | 94.0 ± 18.9 | 119.4 ± 41.0 | 94.8 ± 10.5 |
|  | ALP (U/L) | 39.8 ± 7.6 | 36.2 ± 4.0 | 36.0 ± 9.3 | 39.1 ± 10.2 |
|  | CK (U/L) | 298 ± 116 | 308 ± 127 | 332 ± 66 | 287 ± 140 |
|  | TBIL (μmol/L) | 0.760 ± 0.594 | 0.460 ± 0.167 | 0.840 ± 0.527 | 0.300 ± 0.265 |
|  | TP (g/L) | 69.2 ± 6.0 | 70.8 ± 4.2 | 69.8 ± 5.9 | 70.3 ± 6.0 |
|  | ALB (g/L) | 34.5 ± 2.2 | 35.8 ± 1.9 | 35.7 ± 2.6 | 35.8 ± 2.8 |
|  | GLU (mmol/L) | 7.59 ± 0.56 | 6.04 ± 2.81 | 7.18 ± 0.84 | 7.12 ± 0.60 |
|  | UREA (mmol/L) | 6.00 ± 0.50 | 5.26 ± 0.59 | 6.30 ± 0.61 | 6.04 ± 1.04 |
|  | CRE (μmol/L) | 51.6 ± 4.3 | 46.8 ± 2.6 | 48.7 ± 5.3 | 47.5 ± 6.6 |
|  | TCHO (mmol/L) | 2.064 ± 0.219 | 1.868 ± 0.685 | 2.212 ± 0.330 | 2.222 ± 0.572 |
|  | TG (mmol/L) | 0.494 ± 0.277 | 0.628 ± 0.155 | 0.558 ± 0.196 | 0.589 ± 0.244 |
|  | GGT (U/L) | 0.330 ± 0.152 | 0.336 ± 0.166 | 0.446 ± 0.117 | 0.344 ± 0.178 |
|  | K^+^ (mmol/L) | 3.12 ± 0.10 | 3.32 ± 0.14 | 3.21 ± 0.17 | 3.30 ± 0.11 |
|  | Na^+^ (mmol/L) | 137 ± 2 | 136 ± 1 | 136 ± 1 | 136 ± 1 |
|  | Cl^+^ (mmol/L) | 99.2 ± 0.7 | 100.3 ± 0.4 | 99.2 ± 1.9 | 99.0 ± 3.0 |

Data are expressed as mean ± SD with one-way ANOVA followed by the LSD multiple comparisons test, statistically significant compared to control (^*^*P* 0.05, ^**^*P* < 0.01, ^***^*P* < 0.001; D91 *n* = 5, D182 *n* = 10, D210 *n* = 5)
