# Supplementary material for: Integration of transcriptomics and metabolomics reveals toxicological mechanisms of ZhuRiHeng drop pill in the 180-day repeated oral toxicity study
Source: Front Pharmacol. 2024 Mar 15;15:1333167. doi: 10.3389/fphar.2024.1333167 (PMC10978746; doi:10.3389/fphar.2024.1333167)
Supplement: Supplementary file 3 [file Table3.DOC]

**Table S3.** Hematological analysis of male SD rats during 180-day repeated oral toxicity study.

| Time  point | Parameters | Groups | | | |
| --- | --- | --- | --- | --- | --- |
|  |  | Control | 0.934 g/kg | 1.868 g/kg | 3.736 g/kg |
| D91  (mid-dosing period) | RBC (×10^12^/L) | 8.62 ± 0.27 | 8.49 ± 0.27 | 8.27 ± 0.48 | 8.48 ± 0.27 |
|  | HCT (%) | 41.9 ± 1.8 | 41.9 ± 1.7 | 41.0 ± 0.6 | 42.7 ± 1.3 |
|  | MCV (fL) | 48.7 ± 0.7 | 49.3 ± 1.9 | 49.8 ± 2.4 | 50.4 ± 0.5 |
|  | HGB (g/L) | 149 ± 7 | 147 ± 6 | 143 ± 2 | 150 ± 3 |
|  | MCH (pg) | 17.3 ± 0.3 | 17.4 ± 0.7 | 17.3 ± 0.8 | 17.8 ± 0.4 |
|  | MCHC (g/L) | 356 ± 4 | 352 ± 3 | 348 ± 3^**^ | 352 ± 4 |
|  | PLT (×10^9^/L) | 877 ± 46 | 872 ± 85 | 876 ± 138 | 814 ± 140 |
|  | WBC (×10^9^/L) | 3.88 ± 0.76 | 4.17 ± 1.15 | 4.23 ± 1.21 | 4.77 ± 1.45 |
|  | LYM % | 71.0 ± 8.9 | 73.8 ± 5.9 | 70.6 ± 5.5 | 74.6 ± 9.2 |
|  | NEUT % | 25.2 ± 8.3 | 22.0 ± 6.6 | 24.1 ± 5.1 | 21.5 ± 8.6 |
|  | EOS % | 1.920 ± 0.409 | 2.100 ± 0.534 | 2.725 ± 0.310^*^ | 1.540 ± 0.611 |
|  | BASO % | 0.000 ± 0.000 | 0.000 ± 0.000 | 0.000 ± 0.000 | 0.000 ± 0.000 |
|  | MONO % | 1.94 ± 0.60 | 2.06 ± 0.86 | 2.55 ± 1.01 | 2.38 ± 0.61 |
|  | RET % | 2.77 ± 0.54 | 3.02 ± 0.32 | 3.31 ± 0.37 | 3.40 ± 0.47 |
|  | PT (s) | 19.5 ± 2.3 | 19.2 ± 1.2 | 18.9 ± 0.4 | 20.2 ± 0.7 |
|  | APTT (s) | 20.4 ± 1.0 | 19.0 ± 1.2 | 19.0 ± 0.9 | 19.2 ± 0.6 |
|  | TT (s) | 25.6 ± 2.4 | 27.9 ± 0.8 | 25.8 ± 0.6 | 27.4 ± 1.2 |
|  | FIB (g/L) | 1.86 ± 0.03 | 1.78 ± 0.07 | 1.74 ± 0.05 | 1.78 ± 0.13 |
| D183  (end-dosing period) | RBC (×10^12^/L) | 8.39 ± 0.55 | 8.57 ± 0.38 | 8.34 ± 0.23 | 8.57 ± 0.27 |
|  | HCT (%) | 40.1 ± 1.5 | 40.6 ± 1.4 | 40.0 ± 1.5 | 42.0 ± 1.3^**^ |
|  | MCV (fL) | 47.9 ± 2.1 | 47.4 ± 1.5 | 47.9 ± 1.4 | 49.0 ± 1.8 |
|  | HGB (g/L) | 140 ± 7 | 143 ± 7 | 141 ± 5 | 148 ± 5^**^ |
|  | MCH (pg) | 16.8 ± 0.5 | 16.7 ± 0.5 | 16.9 ± 0.6 | 17.3 ± 0.6 |
|  | MCHC (g/L) | 350 ± 6 | 353 ± 8 | 351 ± 6 | 352 ± 6 |
|  | PLT (×10^9^/L) | 861 ± 96 | 940 ± 108 | 962 ± 67 | 968 ± 98 |
|  | WBC (×10^9^/L) | 4.65 ± 1.37 | 4.51 ± 1.61 | 4.60 ± 0.82 | 6.20 ± 3.11 |
|  | LYM % | 65.7 ± 7.5 | 68.0 ± 6.8 | 65.2 ± 7.5 | 72.0 ± 4.0 |
|  | NEUT % | 27.3 ± 7.2 | 25.8 ± 7.0 | 28.2 ± 7.4 | 22.3 ± 3.9 |
|  | EOS % | 2.540 ± 0.665 | 2.200 ± 0.849 | 2.320 ± 0.581 | 2.120 ± 0.625 |
|  | BASO % | 0.000 ± 0.000 | 0.000 ± 0.000 | 0.000 ± 0.000 | 0.000 ± 0.000 |
|  | MONO % | 4.51 ± 1.19 | 4.01 ± 1.25 | 4.25 ± 1.46 | 3.61 ± 1.23 |
|  | RET % | 3.47 ± 0.49 | 3.27 ± 0.38 | 3.00 ± 0.52 | 3.29 ± 0.78 |
|  | PT (s) | 19.7 ± 1.4 | 19.0 ± 2.1 | 17.3 ± 1.8 | 18.7 ± 2.7 |
|  | APTT (s) | 20.2 ± 1.6 | 20.8 ± 2.8 | 20.5 ± 1.8 | 21.2 ± 2.3 |
|  | TT (s) | 23.9 ± 0.8 | 23.2 ± 1.3 | 23.0 ± 1.1 | 22.8 ± 1.2 |
|  | FIB (g/L) | 1.50 ± 0.15 | 1.55 ± 0.14 | 1.52 ± 0.22 | 1.52 ± 0.21 |
| D210  (recovery period) | RBC (×10^12^/L) | 8.79 ± 0.41 | 8.94 ± 0.34 | 8.76 ± 0.27 | 8.77 ± 0.45 |
|  | HCT (%) | 42.7 ± 2.5 | 43.2 ± 1.5 | 42.7 ± 1.2 | 42.9 ± 2.5 |
|  | MCV (fL) | 48.6 ± 1.4 | 48.4 ± 2.1 | 48.8 ± 1.7 | 48.9 ± 1.5 |
|  | HGB (g/L) | 146 ± 8 | 148 ± 5 | 146 ± 6 | 149 ± 9 |
|  | MCH (pg) | 16.6 ± 0.7 | 16.6 ± 0.7 | 16.7 ± 0.6 | 17.0 ± 0.5 |
|  | MCHC (g/L) | 341 ± 7 | 342 ± 6 | 342 ± 5 | 348 ± 1 |
|  | PLT (×10^9^/L) | 853 ± 36 | 924 ± 100 | 897 ± 59 | 979 ± 91 |
|  | WBC (×10^9^/L) | 4.41 ± 1.19 | 4.32 ± 0.92 | 5.89 ± 2.02 | 4.42 ± 1.20 |
|  | LYM % | 71.2 ± 5.6 | 68.4 ± 8.2 | 68.8 ± 15.5 | 69.0 ± 8.3 |
|  | NEUT % | 22.84 ± 5.35 | 24.58 ± 7.68 | 25.36 ± 14.28 | 22.58 ± 8.25 |
|  | EOS % | 2.18 ± 0.53 | 2.66 ± 0.74 | 2.00 ± 0.52 | 1.94 ± 0.59 |
|  | BASO % | 0.000 ± 0.000 | 0.000 ± 0.000 | 0.000 ± 0.000 | 0.000 ± 0.000 |
|  | MONO % | 3.78 ± 1.16 | 4.36 ± 1.65 | 3.80 ± 1.54 | 6.50 ± 2.72 |
|  | RET % | 3.01 ± 0.24 | 2.89 ± 0.82 | 3.06 ± 0.39 | 3.13 ± 0.72 |
|  | PT (s) | 19.0 ± 1.5 | 19.4 ± 1.4 | 19.6 ± 0.8 | 20.0 ± 0.7 |
|  | APTT (s) | 20.4 ± 1.6 | 20.4 ± 1.8 | 20.2 ± 1.4 | 20.7 ± 1.4 |
|  | TT (s) | 25.5 ± 0.7 | 25.5 ± 0.5 | 25.8 ± 0.8 | 25.0 ± 0.8 |
|  | FIB (g/L) | 1.58 ± 0.13 | 1.63 ± 0.13 | 1.60 ± 0.08 | 1.64 ± 0.18 |

Data are expressed as mean ± SD with one-way ANOVA followed by the LSD multiple comparisons test, statistically significant compared to control (^*^*P* < 0.05, ^**^*P* < 0.01, ^***^*P* < 0.001; D91 *n* = 5 (Control Group, 0.934 g/kg, 3.736 g/kg), *n* = 4 (1.868 g/kg) D183 *n* = 10, D210 *n* = 5)
