# Supplementary material for: Integration of transcriptomics and metabolomics reveals toxicological mechanisms of ZhuRiHeng drop pill in the 180-day repeated oral toxicity study
Source: Front Pharmacol. 2024 Mar 15;15:1333167. doi: 10.3389/fphar.2024.1333167 (PMC10978746; doi:10.3389/fphar.2024.1333167)
Supplement: Supplementary file 4 [file Table1.DOC]

**Table S1.** ECG parameters of male SD rats during 180-day repeated oral toxicity study.

| Time  point | Parameters | Groups | | | |
| --- | --- | --- | --- | --- | --- |
|  |  | Control | 0.934 g/kg | 1.868 g/kg | 3.736 g/kg |
| D91  (mid-dosing period) | Pa (mv) | 5.400 ± 3.130 | 2.000 ± 5.339 | 2.600 ± 2.408 | 2.800 ± 1.643 |
|  | Q (mv) | −0.800 ± 1.789 | 0.000 ± 0.000 | −1.600 ± 2.191 | −1.600 ± 3.578 |
|  | R (mv) | 26.00 ± 6.24 | 30.00 ± 13.29 | 27.00 ± 13.44 | 20.80 ± 13.37 |
|  | S (mv) | −51.800 ± 12.578 | −56.200 ± 19.305 | −56.200 ± 28.341 | −54.800 ± 9.338 |
|  | ST (mv) | −16.200 ± 7.463 | −20.000 ± 10.271 | −17.600 ± 12.033 | −17.000 ± 6.164 |
|  | T (mv) | 4.200 ± 6.419 | 4.800 ± 10.060 | 5.000 ± 8.689 | −6.400 ± 4.393 |
|  | Pd (ms) | 15.200 ± 8.786 | 12.400 ± 8.081 | 10.800 ± 10.756 | 11.200 ± 7.759 |
|  | QRS (ms) | 20.8 ± 2.5 | 17.8 ± 2.7 | 20.6 ± 3.0 | 20.6 ± 2.1 |
|  | RR (ms) | 161 ± 21 | 175 ± 21 | 238 ± 137 | 190 ± 47 |
|  | PR (ms) | 42.800 ± 24.263 | 38.200 ± 21.649 | 33.600 ± 30.778 | 38.600 ± 21.617 |
|  | QT (ms) | 30.200 ± 27.644 | 62.000 ± 15.067 | 40.000 ± 23.033 | 32.400 ± 31.358 |
|  | HR (times/min) | 378 ± 52 | 347 ± 43 | 302 ± 113 | 329 ± 64 |
| D183  (end-dosing period) | Pa (mv) | 3.400 ± 3.565 | 5.100 ± 3.784 | 5.100 ± 2.685 | 3.400 ± 6.077 |
|  | Q (mv) | 0.000 ± 0.000 | 0.000 ± 0.000 | 0.000 ± 0.000 | −1.800 ± 3.795 |
|  | R (mv) | 31.90 ± 17.41 | 37.20 ± 8.40 | 39.30 ± 9.86 | 45.90 ± 18.83 |
|  | S (mv) | −35.400 ± 23.196 | −35.500 ± 15.190 | −30.700 ± 10.382 | −26.600 ± 14.759 |
|  | ST(mv) | −7.700 ± 11.917 | −2.300 ± 13.267 | 1.400 ± 8.934 | 7.500 ± 22.397 |
|  | T (mv) | 3.300 ± 5.889 | 7.900 ± 4.228 | 4.800 ± 6.663 | 2.900 ± 13.085 |
|  | Pd (ms) | 14.100 ± 8.711 | 16.600 ± 2.591 | 15.500 ± 6.276 | 17.100 ± 6.871 |
|  | QRS (ms) | 18.6 ± 2.2 | 19.6 ± 3.3 | 20.1 ± 2.3 | 21.8 ± 4.5 |
|  | RR (ms) | 186 ± 22 | 176 ± 13 | 194 ± 8 | 189 ± 36 |
|  | PR (ms) | 37.500 ± 20.184 | 53.000 ± 5.518 | 49.700 ± 18.512 | 45.700 ± 17.525 |
|  | QT (ms) | 55.600 ± 20.876 | 58.700 ± 22.116 | 55.100 ± 22.811 | 62.800 ± 26.849 |
|  | HR ( times/min) | 326 ± 40 | 342 ± 25 | 310 ± 12 | 328 ± 66 |
| D210  (recovery period) | Pa (mv) | 4.000 ± 2.915 | 4.600 ± 1.140 | 4.200 ± 7.596 | 7.000 ± 2.345 |
|  | Q (mv) | −0.600 ± 1.342 | 0.000 ± 0.000 | 0.000 ± 0.000 | −0.800 ± 1.789 |
|  | R (mv) | 44.0 ± 2.0 | 40.4 ± 16.1 | 41.4 ± 12.3 | 35.0 ± 9.9 |
|  | S (mv) | −28.400 ± 5.030 | −24.600 ± 19.832 | −24.200 ± 11.692 | −20.600 ± 6.309 |
|  | ST (mv) | −0.600 ± 2.074 | −0.600 ± 8.019 | −0.600 ± 7.232 | 5.600 ± 7.092 |
|  | T (mv) | 3.000 ± 5.196 | 0.600 ± 6.950 | 7.400 ± 5.899 | 1.400 ± 10.065 |
|  | Pd (ms) | 13.800 ± 8.012 | 16.200 ± 1.924 | 18.200 ± 3.114 | 17.600 ± 2.302 |
|  | QRS (ms) | 17.4 ± 1.5 | 18.2 ± 2.6 | 21.2 ± 1.9 | 20.2 ± 6.7 |
|  | RR (ms) | 190 ± 23 | 206 ± 23 | 206 ± 28 | 194 ± 9 |
|  | PR (ms) | 42.800 ± 24.160 | 56.400 ± 0.894 | 53.400 ± 5.595 | 56.400 ± 4.393 |
|  | QT (ms) | 55.2 ± 9.7 | 54.6 ± 9.0 | 66.4 ± 16.7 | 58.8 ± 6.8 |
|  | HR (times/min) | 321 ± 47 | 294 ± 32 | 295 ± 38 | 309 ± 15 |

Data are expressed as mean ± SD with one-way ANOVA followed by the LSD multiple comparisons test, statistically significant compared to control (^*^*P* < 0.05, ^**^*P* < 0.01, ^***^*P* < 0.001; D91 *n* = 5, D183 *n* = 10, D210 *n* = 5)
