# Supplementary material for: Integration of transcriptomics and metabolomics reveals toxicological mechanisms of ZhuRiHeng drop pill in the 180-day repeated oral toxicity study
Source: Front Pharmacol. 2024 Mar 15;15:1333167. doi: 10.3389/fphar.2024.1333167 (PMC10978746; doi:10.3389/fphar.2024.1333167)
Supplement: Supplementary file 5 [file Table5.DOC]

**Table S5**. Serum biochemistry analysis of male SD rats during 180-day repeated oral toxicity study.

| Time  point | Parameters | Groups | | | |
| --- | --- | --- | --- | --- | --- |
|  |  | Control | 0.934 g/kg | 1.868 g/kg | 3.736 g/kg |
| D91  (mid-dosing period) | ALT (U/L) | 60.4 ± 43.8 | 33.6 ± 5.9 | 33.3 ± 6.2 | 37.0 ± 16.8 |
|  | AST (U/L) | 142.0 ± 83.9 | 114.4 ± 20.0 | 120.3 ± 13.3 | 111.4 ± 26.7 |
|  | ALP (U/L) | 113.0 ± 17.2 | 102.0 ± 11.5 | 96.3 ± 23.5 | 96.4 ± 12.1 |
|  | CK (U/L) | 483 ± 122 | 550 ± 112 | 585 ± 143 | 487 ± 159 |
|  | TBIL (μmol/L) | 2.16 ± 2.11 | 1.20 ± 0.16 | 1.30 ± 0.14 | 1.52 ± 0.84 |
|  | TP (g/L) | 53.2 ± 1.7 | 53.3 ± 1.6 | 53.4 ± 1.4 | 51.7 ± 2.9 |
|  | ALB (g/L) | 30.7 ± 0.8 | 31.1 ± 1.2 | 31.5 ± 1.0 | 30.3 ± 1.4 |
|  | GLU (mmol/L) | 6.87 ± 0.65 | 6.74 ± 0.42 | 6.26 ± 0.66 | 5.96 ± 0.67 |
|  | UREA (mmol/L) | 6.80 ± 0.83 | 6.58 ± 1.01 | 6.03 ± 0.88 | 7.26 ± 0.92 |
|  | CRE (μmol/L) | 44.0 ± 3.7 | 46.8 ± 7.6 | 43.3 ± 3.6 | 45.2 ± 11.1 |
|  | TCHO (mmol/L) | 1.58 ± 0.23 | 1.61 ± 0.35 | 1.76 ± 0.11 | 1.62 ± 0.18 |
|  | TG (mmol/L) | 0.458 ± 0.339 | 0.940 ± 0.416^*^ | 0.803 ± 0.111 | 0.418 ± 0.247 |
|  | GGT (U/L) | 0.458 ± 0.159 | 0.788 ± 1.048 | 0.370 ± 0.324 | 0.418 ± 0.136 |
|  | K^+^ (mmol/L) | 4.11 ± 0.19 | 4.30 ± 0.14 | 4.23 ± 0.27 | 4.31 ± 0.49 |
|  | Na^+^ (mmol/L) | 146 ± 1 | 145 ± 1 | 146 ± 1 | 146 ± 2 |
|  | Cl^+^ (mmol/L) | 106 ± 1 | 106 ± 1 | 106 ± 1 | 107 ± 1 |
| D183  (end-dosing period) | ALT (U/L) | 43.0 ± 8.6 | 41.9 ± 15.4 | 31.7 ± 3.9 | 41.4 ± 9.6 |
|  | AST (U/L) | 119.5 ± 23.5 | 105.8 ± 31.4 | 123.1 ± 29.7 | 120.1 ± 34.5 |
|  | ALP (U/L) | 108.8 ± 23.1 | 107.6 ± 25.4 | 92.6 ± 18.2 | 104.5 ± 26.7 |
|  | CK (U/L) | 392.7 ± 157.8 | 346.8 ± 213.0 | 533.6 ± 272.9 | 481.2 ± 281.0 |
|  | TBIL (μmol/L) | 0.540 ± 0.272 | 0.530 ± 0.250 | 0.480 ± 0.274 | 0.540 ± 0.337 |
|  | TP (g/L) | 63.3 ± 1.9 | 64.5 ± 2.5 | 62.5 ± 2.0 | 62.9 ± 2.6 |
|  | ALB (g/L) | 31.7 ± 1.1 | 32.1 ± 0.9 | 31.2 ± 0.8 | 31.8 ± 0.9 |
|  | GLU (mmol/L) | 7.72 ± 0.78 | 7.78 ± 0.67 | 7.06 ± 0.98 | 6.85 ± 0.35^*^ |
|  | UREA (mmol/L) | 6.15 ± 0.91 | 5.63 ± 0.89 | 6.62 ± 1.36 | 6.79 ± 0.89 |
|  | CRE (μmol/L) | 42.8 ± 3.9 | 39.1 ± 5.4 | 40.1 ± 4.6 | 40.9 ± 4.4 |
|  | TCHO (mmol/L) | 1.54 ± 0.21 | 1.80 ± 0.19 | 1.78 ± 0.27 | 1.77 ± 0.30 |
|  | TG (mmol/L) | 0.731 ± 0.330 | 0.960 ± 0.389 | 0.666 ± 0.109 | 1.051 ± 0.543 |
|  | GGT (U/L) | 0.261 ± 0.158 | 0.284 ± 0.108 | 0.335 ± 0.122 | 0.388 ± 0.171 |
|  | K^+^ (mmol/L) | 4.01 ± 0.11 | 4.19 ± 0.18^*^ | 4.17 ± 0.27 | 4.33 ± 0.17^***^ |
|  | Na^+^ (mmol/L) | 146 ± 2 | 145 ± 1 | 144 ± 1 | 145 ± 1 |
|  | Cl^+^ (mmol/L) | 107 ± 1 | 107 ± 1 | 106 ± 2 | 107 ± 1 |
| D210  (recovery period) | ALT (U/L) | 51.2 ± 5.7 | 42.0 ± 4.6 | 62.6 ± 22.8 | 45.4 ± 4.2 |
|  | AST (U/L) | 110.8 ± 33.8 | 123.4 ± 34.2 | 146.4 ± 40.9 | 121.4 ± 18.4 |
|  | ALP (U/L) | 93.0 ± 22.5 | 99.0 ± 17.6 | 97.2 ± 43.2 | 63.6 ± 13.9 |
|  | CK (U/L) | 510 ± 360 | 571 ± 283 | 606 ± 314 | 861 ± 341 |
|  | TBIL (μmol/L) | 0.700 ± 0.141 | 0.960 ± 0.261 | 1.140 ± 0.757 | 0.860 ± 0.270 |
|  | TP (g/L) | 63.7 ± 2.3 | 62.8 ± 2.1 | 62.3 ± 1.7 | 61.3 ± 3.2 |
|  | ALB (g/L) | 31.7 ± 0.7 | 31.3 ± 0.9 | 30.9 ± 1.0 | 30.7 ± 1.7 |
|  | GLU (mmol/L) | 7.42 ± 0.40 | 7.23 ± 1.01 | 7.29 ± 1.24 | 6.58 ± 0.95 |
|  | UREA (mmol/L) | 5.08 ± 0.63 | 4.74 ± 0.44 | 5.66 ± 0.75 | 5.04 ± 0.76 |
|  | CRE (μmol/L) | 39.9 ± 3.2 | 35.3 ± 5.0 | 42.3 ± 6.8 | 36.4 ± 4.7 |
|  | TCHO (mmol/L) | 1.65 ± 0.22 | 1.89 ± 0.31 | 1.88 ± 0.19 | 1.73 ± 0.23 |
|  | TG (mmol/L) | 1.202 ± 0.787 | 1.568 ± 1.071 | 0.848 ± 0.397 | 1.998 ± 1.193 |
|  | GGT (U/L) | 0.396 ± 0.178 | 0.352 ± 0.096 | 0.628 ± 0.346 | 0.466 ± 0.098 |
|  | K^+^ (mmol/L) | 3.59 ± 0.26 | 3.68 ± 0.15 | 3.83 ± 0.36 | 3.85 ± 0.18 |
|  | Na^+^ (mmol/L) | 137 ± 1 | 137 ± 1 | 137 ± 2 | 137 ± 1 |
|  | Cl^+^ (mmol/L) | 99.3 ± 0.3 | 99.6 ± 1.7 | 100.3 ± 2.3 | 100.4 ± 2.5 |

Data are expressed as mean ± SD with one-way ANOVA followed by the LSD multiple comparisons test, statistically significant compared to control (^*^*P* < 0.05, ^**^*P* < 0.01, ^***^*P* < 0.001; D91 *n* = 5 (Control Group, 0.934 g/kg, 3.736 g/kg), *n* = 4 (1.868 g/kg), D183 *n* = 10, D210 *n* = 5)
