# Supplementary material for: Integration of transcriptomics and metabolomics reveals toxicological mechanisms of ZhuRiHeng drop pill in the 180-day repeated oral toxicity study
Source: Front Pharmacol. 2024 Mar 15;15:1333167. doi: 10.3389/fphar.2024.1333167 (PMC10978746; doi:10.3389/fphar.2024.1333167)
Supplement: Supplementary file 8 [file Table2.DOC]

**Table S2.** ECG parameters of female SD rats during 180-day repeated oral toxicity study.

| Time  point | Parameters | Groups | | | |
| --- | --- | --- | --- | --- | --- |
|  |  | Control | 0.934 g/kg | 1.868 g/kg | 3.736 g/kg |
| D91  (mid-dosing period) | Pa (mv) | −0.200 ± 5.586 | 4.000 ± 2.550 | −0.600 ± 9.290 | 5.200 ± 3.114 |
|  | Q (mv) | 0.000 ± 0.000 | 0.000 ± 0.000 | −0.600 ± 1.342 | 0.000 ± 0.000 |
|  | R (mv) | 44.8 ± 20.0 | 36.6 ± 14.0 | 61.4 ± 19.8 | 44.2 ± 16.4 |
|  | S (mv) | −33.600 ± 11.718 | −66.600 ± 31.254 | −66.200 ± 35.731 | −75.800 ± 30.310 |
|  | ST (mv) | −6.200 ± 6.870 | −22.400 ± 14.328 | −21.400 ± 21.709 | −21.600 ± 17.473 |
|  | T (mv) | 0.200 ± 11.649 | −3.600 ± 11.824 | 0.600 ± 4.722 | 4.000 ± 10.416 |
|  | Pd (ms) | 13.600 ± 8.295 | 13.400 ± 7.861 | 27.400 ± 3.847^**^ | 14.000 ± 8.031 |
|  | QRS (ms) | 17.6 ± 2.2 | 17.8 ± 2.7 | 22.2 ± 4.2 | 19.4 ± 3.3 |
|  | RR (ms) | 163 ± 9 | 173 ± 10 | 179 ± 20 | 179 ± 13 |
|  | PR (ms) | 34.600 ± 20.256 | 34.000 ± 20.433 | 46.000 ± 5.745 | 39.400 ± 22.052 |
|  | QT (ms) | 67.800 ± 14.481 | 66.200 ± 37.539 | 59.400 ± 6.542 | 60.400 ± 33.990 |
|  | HR (times/min) | 369 ± 21 | 349 ± 20 | 339 ± 38 | 336 ± 23 |
| D182  (end-dosing period) | Pa (mv) | 5.700 ± 4.523 | 4.800 ± 6.356 | 8.200 ± 2.530 | 6.500 ± 2.953 |
|  | Q (mv) | 0.000 ± 0.000 | 0.000 ± 0.000 | 0.000 ± 0.000 | 0.000 ± 0.000 |
|  | R (mv) | 67.6 ± 21.1 | 66.4 ± 14.7 | 68.2 ± 13.2 | 72.4 ± 13.5 |
|  | S (mv) | −40.600 ± 8.822 | −32.100 ± 14.106 | −30.900 ± 18.126 | −36.000 ± 16.560 |
|  | ST (mv) | 2.600 ± 8.316 | 9.100 ± 8.171 | 12.900 ± 17.156 | 9.900 ± 15.118 |
|  | T (mv) | 10.100 ± 4.771 | 12.300 ± 5.794 | 15.400 ± 7.531 | 16.200 ± 5.095 |
|  | Pd (ms) | 18.700 ± 5.208 | 19.300 ± 13.590 | 16.200 ± 3.425 | 16.100 ± 6.154 |
|  | QRS(ms) | 19.1 ± 3.2 | 19.8 ± 2.5 | 19.8 ± 1.7 | 18.4 ± 1.5 |
|  | RR(ms) | 193 ± 13 | 205 ± 14 | 225 ± 21^**^ | 232 ± 43 |
|  | PR (ms) | 51.100 ± 3.573 | 48.600 ± 19.057 | 54.200 ± 4.392 | 50.900 ± 18.297 |
|  | QT (ms) | 73.1 ± 11.9 | 77.7 ± 13.6 | 80.8 ± 12.4 | 77.7 ± 12.0 |
|  | HR (times/min) | 312 ± 23 | 294 ± 19 | 269 ± 25^**^ | 267 ± 50 |
| D210  (recovery period) | Pa (mv) | 8.00 ± 3.61 | 8.40 ± 1.67 | 6.60 ± 0.89 | 5.40 ± 1.82 |
|  | Q (mv) | 0.000 ± 0.000 | 0.000 ± 0.000 | 0.000 ± 0.000 | 0.000 ± 0.000 |
|  | R (mv) | 54.6 ± 19.6 | 56.8 ± 10.6 | 66.8 ± 12.9 | 58.0 ± 14.8 |
|  | S (mv) | −55.200 ± 19.058 | −32.200 ± 17.796 | −37.800 ± 13.312 | −53.800 ± 20.657 |
|  | ST (mv) | −6.800 ± 15.320 | 2.200 ± 10.616 | 5.200 ± 15.579 | −8.800 ± 19.318 |
|  | T (mv) | 4.600 ± 3.286 | 6.000 ± 10.954 | 11.200 ± 8.871 | 9.200 ± 3.899 |
|  | Pd (ms) | 15.4 ± 3.6 | 17.6 ± 1.8 | 16.2 ± 1.3 | 15.0 ± 2.4 |
|  | QRS (ms) | 22.4 ± 4.3 | 16.6 ± 2.1^**^ | 18.0 ± 3.2^*^ | 20.2 ± 2.0 |
|  | RR (ms) | 176 ± 11 | 185 ± 24 | 204 ± 10^*^ | 215 ± 33 |
|  | PR (ms) | 50.6 ± 3.6 | 51.0 ± 1.6 | 52.4 ± 2.9 | 51.8 ± 4.7 |
|  | QT (ms) | 8.00 ± 3.61 | 8.40 ± 1.67 | 6.60 ± 0.89 | 5.40 ± 1.82 |
|  | HR (times/min) | 343 ± 24 | 329 ± 40 | 294 ± 15^*^ | 285 ± 45 |

Data are expressed as mean ± SD with one-way ANOVA followed by the LSD multiple comparisons test, statistically significant compared to control (^*^*P* < 0.05, ^**^*P* < 0.01, ^***^*P* < 0.001; D91 *n* = 5, D182 *n* = 10, D210 *n* = 5
